# Design Criteria for Architected Materials with Programmable Mechanical Properties Within Theoretical Limit Ranges

**DOI:** 10.1002/advs.202307279

**Published:** 2023-12-12

**Authors:** Peng Yin, Baotong Li, Jun Hong, Hui Jing, Bang Li, Honglei Liu, Xiaoming Chen, Yang Lu, Jinyou Shao

**Affiliations:** ^1^ Key Laboratory of Education Ministry for Modern Design and Rotor‐Bearing System Xi'an Jiaotong University Xi'an Shaanxi 710049 China; ^2^ State Key Laboratory for Manufacturing Systems Engineering Xi'an Jiaotong University Xi'an Shaanxi 710049 China; ^3^ Department of Mechanical Engineering The University of Hong Kong Pokfulam Hong Kong SAR 999077 China

**Keywords:** architected materials, design criteria, heterogeneous assembly, mechanical properties, programmable, theoretical limits

## Abstract

Architected materials comprising periodic arrangements of cells have attracted considerable interest in various fields because of their unconventional properties and versatile functionality. Although some better properties may be exhibited when this homogeneous layout is broken, most such studies rely on a fixed material geometry, which limits the design space for material properties. Here, combining heterogeneous and homogeneous assembly of cells to generate tunable geometries, a hierarchically architected material (HAM) capable of significantly enhancing mechanical properties is proposed. Guided by the theoretical model and 745 752 simulation cases, generic design criteria are introduced, including dual screening for unique mechanical properties and careful assembly of specific spatial layouts, to identify the geometry of materials with extreme properties. Such criteria facilitate the potential for unprecedented properties such as Young's modulus at the theoretical limit and tunable positive and negative Poisson's ratios in an ultra‐large range. Therefore, this study opens a new paradigm for materials with extreme mechanical properties.

## Graphical abstract

Inspired by extensive data, this study reveals a rare and significant mechanical property enhancement mechanism. An effective method to achieve mechanical properties at the theoretical limit (including Young's modulus and Poisson's ratio) is given by combining heterogeneous and homogeneous assembly of the cells. Meanwhile, programmable properties and generic criteria bring architected materials closer to real‐world applications.

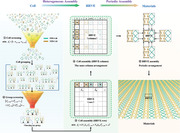

## 1 Introduction

In the study of materials with excellent properties, architected materials, which exhibit unique behavior owing to their geometry rather than composition, have made significant advances in recent years.^[^ ^1^, ^2^, ^3^ ^]^ By rationally engineered material geometries, many exceptional properties, including ultrahigh ratio of stiffness‐weight and strength‐weight,^[^ ^4^, ^5^ ^]^ zero or negative Poisson's ratio,^[^ ^6^, ^7^ ^]^ negative thermal expansion,^[^ ^8^, ^9^ ^]^ target failure load,^[^ ^10^ ^]^ and vanishing shear modulus,^[^ ^11^ ^]^ have been demonstrated. Thus, such materials have been suggested for application in intelligent machines,^[^ ^12^, ^13^ ^]^ shape‐morphing systems,^[^ ^14^, ^15^ ^]^ switchable optical devices,^[^ ^16^, ^17^ ^]^ and stretchable electronics.^[^ ^18^ ^]^ However, most proposed architected materials comprise identical cells arranged periodically, resulting in a single homogeneous deformation response that restricts the material properties to a relatively small range. This underscores the importance of developing strategies for various spatial arrangements to achieve superior properties for the demands of extreme environments.

Heterogeneous assembly provides an ideal platform for the design of superior material properties, because a wealth of deformation responses can be obtained by skillfully integrating different components.^[^ ^19^, ^20^ ^]^ Inspired by the underlying mechanisms,^[^ ^21^, ^22^ ^]^ optimization algorithms,^[^ ^23^, ^24^ ^]^ and biological systems,^[^ ^25^, ^26^, ^27^ ^]^ architected materials assembled using different structures, modules, and even mechanical elements exhibit extraordinary mechanical properties, such as a wide range of Poisson's ratios,^[^ ^28^ ^]^ elastic stiffness at the isotropic limit,^[^ ^29^ ^]^ and extreme strain energy density.^[^ ^30^ ^]^ In addition to material properties, many desirable functionalities can also be achieved through heterogeneous assembly, including continuously tunable mechanical properties,^[^ ^31^, ^32^ ^]^ stable shape memory,^[^ ^33^ ^]^ and geometric reconfiguration.^[^ ^34^ ^]^ Although these cases have shown the potential of heterogeneous assembly to enable excellent properties, they are reliant on fixed material geometry, which renders them suitable for tasks with specific property requirements and limits the design space for material properties. Therefore, there are still ample opportunities for generic design criteria for architected materials with programmable extreme properties.

Here, we introduce a type of hierarchically architected materials (HAMs) based on a combination of heterogeneous and homogeneous assemblies, and show that programmable extreme mechanical properties can be achieved by a rational assembly of different cells with suitable properties. To determine how the cell assembly affects the material properties, the mechanical properties of numerous HAMs were analyzed by simulation. In an effort to obtain HAMs with extreme mechanical properties, a theoretical model capable of accurately evaluating the properties of HAMs was developed and generic design criteria for material geometries with extreme properties were proposed accordingly. Following these criteria, architected materials with an extreme Young's modulus were designed. With a deeper understanding of the criteria, the potential for certain unprecedented properties was demonstrated, including i) Young's modulus at the theoretical limit, ii) tunable positive and negative Poisson's ratios over an ultra‐large range, and iii) both excellent Young's modulus and Poisson's ratio. Thus, our study opens new avenues for the design of architected materials with programmable extreme mechanical properties, and the generic criteria with geometry‐independent features bring such materials closer to real‐world applications.

## 2 Results and Discussion

### 2.1 Design strategy

**Figure 1:**
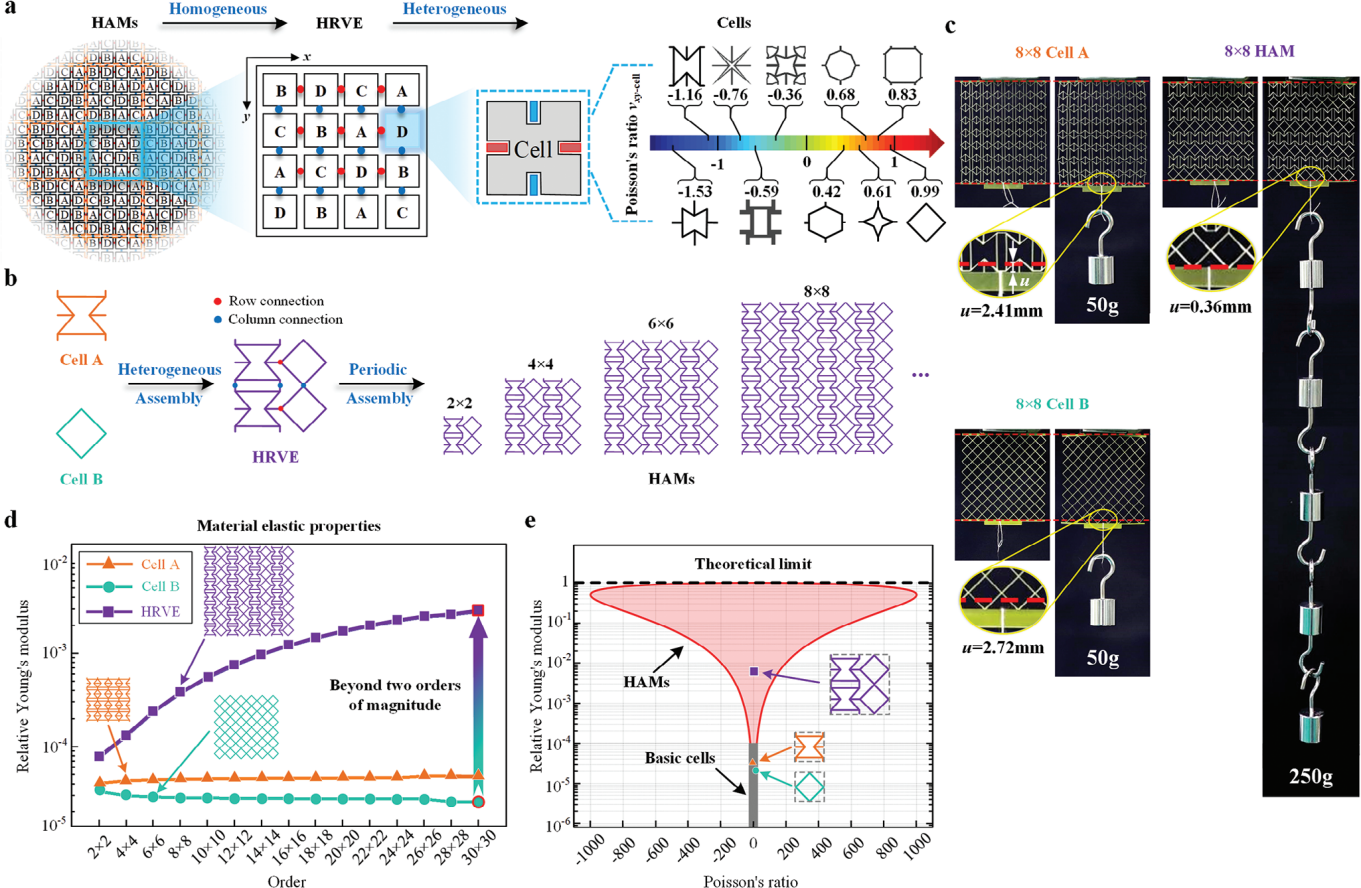
Design strategies to construct HAMs. a) Design strategy of HAMs, including the heterogeneous assembly of cells and the homogeneous assembly of HRVEs. b) Construction processing of HAMs. c) Testing of the enhancement effect of assembly strategy on Young's modulus. d) Plot of relative Young's modulus versus order for HAMs and basic cells (simulation data). e) Elastic properties of HAMs designed based on generic criteria (theoretical data). The red and gray areas denote the properties of the HAMs and the basic cells, respectively. The black dashed line indicates the Young's modulus of the constituent solid materials. The gray dashed lines around material geometries denote the periodic boundary conditions (PBCs).

**Figure** **1**a shows a type of architected materials with clear hierarchical features, referred to as hierarchically architected materials (HAMs). In terms of structural composition, HAMs are divided into three layers: the material, heterogeneous representative volume element (HRVE), and cell layers. The material was formed by the periodic assembly of HRVEs. To achieve extreme mechanical properties (referred to Young's modulus and Poisson's ratio), the conventional homogeneous layout of architected materials needs to be broken.^[^ ^20^ ^]^ Thus, the HRVEs are constructed by assembling cells with different mechanical properties. Specifically, cells with positive and negative Poisson's ratios (i.e., PPR and NPR, which represent deformation patterns in opposite directions^[^ ^6^ ^]^) are used to generate diverse tunable geometries and assembled by the connection points at the boundaries (red and blue points denote row and column connections, respectively). Based on the analysis of the numerical simulation data of numerous HAMs (a total of 745752 cases, including 347988 and 397764 cases in Numerical Experiment I and II, respectively), we found that a few HAMs exerted an extremely significant enhancement effect on the mechanical properties (only two cases in Numerical Experiment I exhibited more than two orders of magnitude enhancement in the Young's modulus) compared to homogeneous materials composed of a single cell (further details in Section 2.2). To realize the forward design of such materials with extreme properties, a theoretical model capable of accurately evaluating the mechanical properties of HAMs was developed. In this theoretical model, the requirements of extreme material properties on the assembled cell properties and cell assembly patterns were determined. Specifically, the requirements for cell property include: i) the Poisson's ratio product of the cells tending to 1 to the best extent possible (i.e., 1 − ν_*yx* − cell_ν_*xy* − cell_ → 0), and ii) the assembled cells containing at least one cell with PPR and one cell with NPR. The requirements for cell assembly include: i) the number ratio of the cells on the rows in the HRVEs that satisfies the extreme property requirements in the model; ii) the same geometry of the cells on columns in HRVEs, iii) the periodic arrangement of the HRVEs, further explanations are provided in Section 2.3. Consequently, the generic criteria for the design of material geometries with extreme mechanical properties, including cell screening and cell assembly criteria, were further proposed according to the above requirements. The cell screening criterion was employed to identify cell geometries that satisfy the requirements of extreme material properties on cell properties, whereas the cell assembly criterion was applied to assemble these screened cell geometries into material geometries (further details in Section 2.4). Next, to verify the effectiveness of the enhancement effect and design criteria, a parametric optimization approach was utilized to construct the cell geometries by optimizing the key geometric parameters (e.g., lengths, angles, and line widths) of the classical architecture (i.e., re‐entrant honeycomb for NPR cells and 4‐STAR system for PPR cells). By applying the two design criteria, HRVEs assembled by cells with unique properties (i.e., ν_*yx* − cell_ ν_*xy* − cell_ =  0.992 for Cell A with NPR and ν_*yx* − cell_ ν_*xy* − cell_ =  0.998 for Cell B with PPR) in specific number ratios (e.g., 1:1) were finally assembled periodically into HAMs with different orders (Figure 1b), see the “Design of HAMs” of Experimental Section for more details. As shown in Figure 1c, compared to the homogeneous material formed by the periodic assembly of a single cell (i.e., 8 × 8 Cell A and 8 × 8 Cell B), the HAM exhibited less displacement (*u*  =  0.36 mm for the 8 × 8 HAM, and *u*  =  2.41 mm for 8 × 8 Cell A and *u*  =  2.72 mm for 8 × 8 Cell B) while bearing more load (250 g weight for the HAM, and 50 g weight for the cells). Thus, the assembly strategy enabled the HAM to have a superior load‐bearing capacity (i.e., a larger Young's modulus). With a further increase in the assembly order (Figure 1d), this property enhancement showed a striking increase (more than two orders of magnitude). Ideally, the Young's modulus at the theoretical limit (i.e., the relative Young's modulus, ${\bar{E}}_{x}=1$, where ${\bar{E}}_{x}=E_{x}/E_{s}$, and *E_x_* and *E_s_* denote the Young's modulus of the HAM and solid constituent material, respectively) would be realized through our criteria (Figure 1e).^[^ ^35^, ^36^ ^]^ More details on the extreme enhancement effects, generic design criteria and unprecedented enhancement potential of HAMs are provided in the following sections.

### 2.2 Enhanced Material Properties

To determine the effect of the assembly strategy on the material properties, we start by analyzing the construction of HAMs to obtain the determinants of the properties. According to the construction principles (Figure 1a), the elastic properties of HAMs are determined by both the basic cells and the HRVE. Because the periodic assembly of HRVEs can be characterized uniformly using a numerical method (i.e., periodic boundary conditions, PBCs), we focused on the effect of cell assembly on the material properties.

In the heterogeneous assembly of cells, cell type and cell number are the important factors for determining the HRVE. Cell types can be distinguished here based on the differences in their elastic properties. Cell number refers to the total number of cells in the HRVE, which is equivalent to the HRVE order. To clarify the effect of different types of cells and their possible pairing patterns on the overall material properties, some representative cells exhibiting a large diversity in elastic properties were carefully chosen (i.e., Young's modulus from isotropy to anisotropy, and Poisson's ratios from PPR to NPR), and cell pairs could be generated from these cells by pairwise combination (Figures S5–S7 for more details, Supporting Information). Because the number of tested HAMs increases exponentially with respect to the HRVE order, the numerical test began from a relatively small‐scale of HRVEs (i.e., 2 × 2 to 4 × 4 orders).

**Figure 2:**
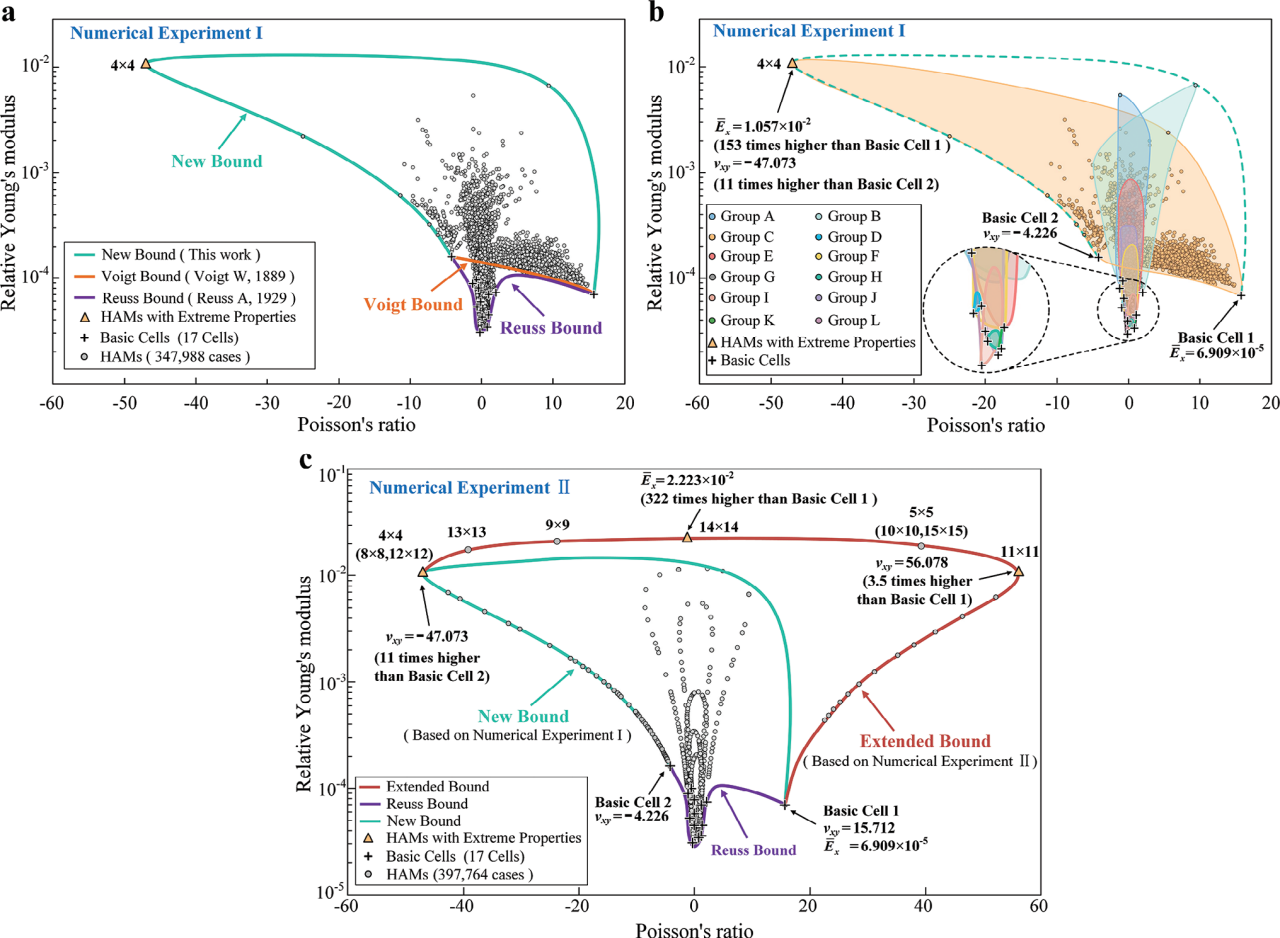
Elastic properties of HAMs in Numerical Experiments. a) Elastic properties of HAMs in Numerical Experiment I (HRVE order from 2 × 2 to 4 × 4). b) Elastic property range of 12 group HAMs in Numerical Experiment I (relative Young's modulus and Poisson's ratio of materials, ${\bar{E}}_{x}$ and ν_*xy*_). c) Elastic properties of HAMs in Numerical Experiment II (HRVE order from 2 × 2 to 15 × 15). In Numerical Experiment II, the HRVE order of the extreme properties is not the largest order. For example, the HRVE order corresponding to the maximum relative Young's modulus is 14 × 14 (Figure 2c), however, the largest set order is 15 × 15. Such HRVE orders are not the same for each group (Figure S10, Supporting Information). Thus, the HRVE order also has an important impact on the material properties.

Excluding the duplicate geometry, 347988 HAMs were assembled from the 12 cell pairs in Numerical Experiment I. Finite element analysis (FEA) was used to evaluate the elastic properties of each HAM. The elastic properties of HAMs are shown in **Figure** **2**a,b. In Figure 2a, a very small number of HAMs exhibited a significant enhancement in elastic properties (marked by yellow triangles). Specifically, compared to the homogeneous materials formed by cells, the marked HAMs exhibited a 153‐fold enhancement in the relative Young's modulus with almost the same material consumption (volume fractions: 3.469% and 3.400% for the homogeneous materials, 3.434% for the HAM). Among the 347988 HAMs, only two cases (0.00057%) showed enhancement effects beyond two orders of magnitude. Such dramatic property enhancements demonstrate that, in rare instances, excellent material properties can be realized through the proposed assembly strategy. Moreover, HAMs with extreme properties enable the new property bound (the New Bound) to be far exceed the classical theory bounds for evaluating the upper (the Voigt Bound^[^ ^37^ ^]^) and lower (the Reuss Bound^[^ ^38^ ^]^) limits of the elastic properties of composites.

**Figure 3:**
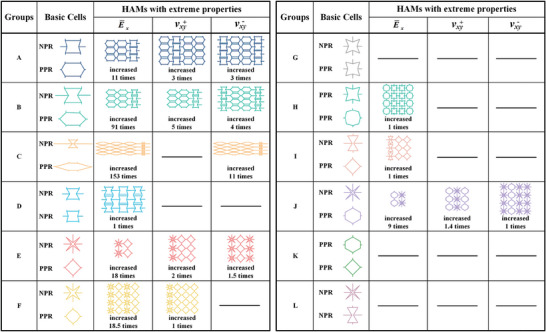
HRVE geometries corresponding to extreme properties in each group. The figure shows the geometries of 12 cell pairs, HRVE geometries of the HAMs with extreme property, and property enhancement multiplier of the HAMs with extreme property in each group compared to the basic cell. Regions without geometry indicate that the HAMs in this group do not exhibit enhancement in material properties corresponding to this region.

Further, the material property bounds for each cell pair are distinguished in Figure 2b. Some bounds exhibited unexpected enhancements in the mechanical properties (e.g., two orders of magnitude enhancement in group C), whereas others never showed similar situations (e.g., no enhancement in groups G, K, and L) (Figure S9, Supporting Information). Interesting, a striking resemblance was observed among the HRVEs corresponding to the extreme properties that emerged in each group, i.e., the cells in each column are of exactly the same geometry (the rows and columns denote the *x*‐ and *y*‐directions of the HRVE, respectively), here referred to as the same column arrangement (**Figure** **3**). Moreover, the property enhancement effect of the HAM proved the validity of the proposed design strategy in that the heterogeneous assembly of positive and negative Poisson's ratio cells can provide a richer deformation response and thus superior material properties. The discovery of the law for the same column arrangement further confirms that extreme material properties require the synergy of homogeneous and heterogeneous assemblies.

As the HRVE orders of the HAMs with extreme properties in each group were not the same, the effect of the HRVE order on the extreme material properties warrants further investigation. Based on the assembly pattern of the same column arrangement, the HRVE order of the original 12 cell pairs was increased to 15 × 15 order in Numerical Experiment II, wherein the number of HAMs was 397764 (Figure S8, Supporting Information). In Figure 2c, as the HRVE order increased, the enhancement of the material properties became more remarkable (322 times in the relative Young's modulus) in the same cell pair assembly (i.e., group C) than that in Numerical Experiment I (153 times). This sustained enhancement suggests that the material properties depend both on the cell type and the HRVE order. Interestingly, the analysis result for the point representing the maximum relative Young's modulus in Figure 2c demonstrates that this point actually represents 182 HAMs with the same properties but different geometries (Figure S11, Supporting Information). More importantly, these 182 HAMs exhibited the surprisingly same number ratio of cells in the HRVE rows (referred to as the row cell ratio), and similar scenarios emerged for other HAMs (Figure S12, Supporting Information). This reveals that the elastic properties of HAMs are correlated with the row cell ratio in HRVEs, rather than the arrangement sequence of cells in the row. Taken together, the simulation results indicate that the proposed HAMs have a significant advantage in terms of mechanical properties, but HAMs with extreme properties are rare. Obviously, the computational cost of using a numerical simulation approach to explore such HAMs is very expensive. Thus, considering this factor and the uncertainty of the property space, a theoretical model capable of evaluating the properties of HAM is required to guide the forward design of materials with extreme properties by determining the appropriate cell types (i.e., basic cells with suitable mechanical properties) and HRVE orders (i.e., the rational assembly of these cells).

### 2.3 Theoretical Model

The establishment of a theoretical model contributes to the identification of the factors influencing material properties through quantitative functional relationships, thereby further enabling the design of materials with superior properties. However, most studies on theoretical models require predetermined geometrical architectures that significantly limit the design space for material geometries and properties. Therefore, developing a theoretical model capable of accurately predicting properties without pre‐defined geometric information is a significant challenge in realizing extreme material properties.

**Figure 4:**
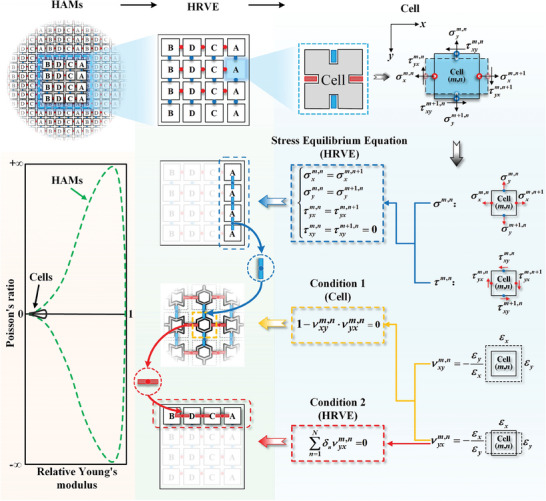
Interpretation of key terms in the theoretical model. These key terms include the stress equilibrium equation for the same column arrangement, condition 1 and 2 that determine the limiting of the mechanical properties of HAMs. The stress equilibrium equations consist of the normal ($\sigma_{x}^{m,n}$ and $\sigma_{y}^{m,n}$) and tangential ($\tau_{xy}^{m,n}$ and $\tau_{yx}^{m,n}$) stresses at the connection points of the (*m*, *n*) cell boundary. Condition 1 affects the material properties by cell properties (i.e., Poisson's ratio in the *x*‐ and *y*‐ directions, $\nu_{xy}^{m,n}$ and $\nu_{yx}^{m,n}$), while condition 2 affects the material properties by both cell geometry (i.e., δ_*n*_) and cell properties (i.e., $\nu_{yx}^{m,n}$). The two conditions correspond to the cell properties and row cell ratios, see Figure 5 for more details. When these two conditions and the same column arrangement pattern are satisfied simultaneously, the design of HAMs with extreme properties would be realized.

Because materials with extreme mechanical properties have the same cell geometry in each column (simulation results), our model focused on handling the elastic property prediction of HAMs with the same column arrangement (**Figure** **4**). According to the definitions of the Young's modulus and Poisson's ratio, the material strain along the *x*‐ and *y*‐directions should first be considered. Using the PBCs, the HAM strains can be calculated by the displacement of the HRVEs. The HRVE displacements were characterized by the displacement differences of the connection points on the HRVE parallel boundary. Owing to the assumptions of the linear elastic model, a linear accumulation was adopted in the construction of the relationship between cell displacement and HRVE displacement. Similar to the HRVE displacement, the cell displacement is defined as the displacement difference between the connection points on the cell parallel boundary. Based on the cell displacement field solved using the stress field, the displacement of any point within the cell can be easily obtained (see ‘Section S1’, Supporting Information for more details). Thus, the key to the evaluation of the HAM properties depends on the solution of the cell stress field.

When a material is subjected to a uniaxial tensile stress *σ_x_* along the *x*‐direction, the stress field of its internal cell changes consequently. The equilibrium equation of the local stresses on the cell boundary, including normal ($\sigma_{x}^{m,n}$ and $\sigma_{y}^{m,n}$) and tangential ($\tau_{xy}^{m,n}$ and $\tau_{yx}^{m,n}$) stresses, was applied for solving the cell stress field (Figure 4). To ensure that the periodic array of HRVEs represents a continuous physical body, two continuities must be satisfied at the boundaries of adjacent HRVEs: i) the displacements must be continuous, and ii) the forces at the relatively parallel boundaries must be the same. Meanwhile, in the case of the same column arrangement, an HRVE with *M* rows and *N* column cells could be equivalent to an HRVE with one row and *N* column cells owing to the PBCs. Consequently, the stress of the connection points on the upper and lower boundaries is the same for all cells in the HRVE row, and there is no tangential stress (further explanations are given in ‘Section S1’, Supporting Information). Thus, the stress equilibrium equation on the cell boundary can be expressed as

(1) $$\left\{\begin{array}{c} \sigma_{x}^{m,n}=\sigma_{x}^{m,n+1}\\ \sigma_{y}^{m,n}=\sigma_{y}^{m+1,n}\\ \tau_{yx}^{m,n}=\tau_{yx}^{m,n+1}\\ \tau_{xy}^{m,n}=\tau_{xy}^{m+1,n}=0 \end{array}\right.$$

When the cell stress field is determined by Equation 1, the Young's modulus *E_x_* and Poisson's ratio ν_*xy*_ of the HAMs in the *x*‐ direction are expressed as

(2) $$E_{x}=\frac{1}{\sum_{n=1}^{N}\frac{\delta_{n}\left(1-\nu_{yx}^{m,n}\nu_{xy}^{m,n}\right)}{E_{x}^{m,n}}+{\left(\sum_{n=1}^{N}\delta_{n}\nu_{yx}^{m,n}\right)}^{2}/\left(\sum_{n=1}^{N}\delta_{n}E_{y}^{m,n}\right)}$$

(3) $$\nu_{xy}=-\frac{\varepsilon_{y}}{\varepsilon_{x}}=\frac{1}{\frac{\left(\sum_{n=1}^{N}\delta_{n}\frac{1-\nu_{yx}^{m,n}\nu_{xy}^{m,n}}{{\bar{E}}_{x}^{m,n}}\right)\sum_{n=1}^{N}\delta_{n}{\bar{E}}_{y}^{m,n}}{\sum_{n=1}^{N}\delta_{n}\nu_{yx}^{m,n}}+\sum_{n=1}^{N}\delta_{n}\nu_{yx}^{m,n}}$$

As shown in Equations (2) and (3), a relatively complex function between the material and cell properties was successfully established (see Figure S13 for more details about the verification of this theoretical model, Supporting Information). This complexity can be demonstrated in two ways. One is that the Young's modulus of HAMs along the *x*‐direction is related to both the Young's modulus of each cell in the *x*‐ and *y*‐ directions (i.e., $E_{x}^{m,n}$ and $E_{y}^{m,n}$), and the Poisson's ratio of the cells along both directions, such as $1-\nu_{yx}^{m,n}\nu_{xy}^{m,n}$. The other is that the geometric parameters of the cell (i.e., δ_*n*_, the length ratio of the cells to the HRVE in the loaded direction) can influence the material properties together with the cell properties, such as $\sum_{n=1}^{N}\delta_{n}\nu_{yx}^{m,n}$. Note that the material distribution within the cells is not constrained in this model. Theoretically, it is possible that the above two terms (i.e., $1-\nu_{yx}^{m,n}\nu_{xy}^{m,n}$ and $\sum_{n=1}^{N}\delta_{n}\nu_{yx}^{m,n}$) are equal to 0. Combined with the fact that both are located in the denominator of Equation 2, they are considered important factors in determining the limit of the Young's modulus of the material. Similarly, the limit of Poisson's ratio also depends on these two parameters (Equation 3). Thus, when HRVEs have the same column arrangement, HAMs with extreme mechanical properties can be obtained by making the cell properties and the row cell ratios (i.e., the number ratio of the basic cells in the HRVE rows) satisfy the above two important conditions (i.e., condition 1: $1-\nu_{yx}^{m,n}\;\nu_{xy}^{m,n}=0$, condition 2: $\sum_{n=1}^{N}\delta_{n}\nu_{yx}^{m,n}=0$) (Figure 4).

### 2.4 Design Criteria

**Figure 5:**
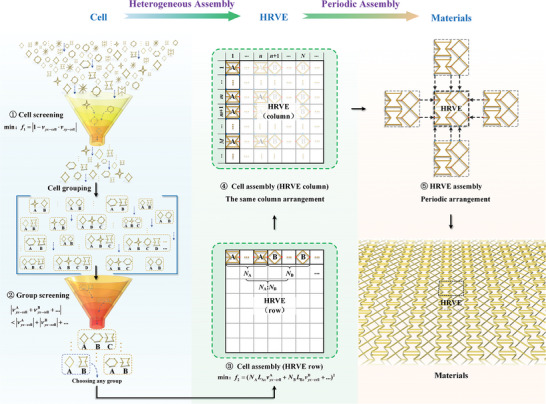
Generic design criteria for HAMs with extreme properties. In the screening criterion, the cells with Poisson's ratio product closest to 1 (the requirement of condition 1) are selected by using minimum:  *f*_1_ = |1 − ν_*yx* − cell_ · ν_*xy* − cell_|  as the objective function of the structure optimization. In the assembly criterion, $\mathrm{minimum}:\;f_{2}={\left(N_{A}L_{Ax}\nu_{yx-\mathrm{cell}}^{A}+N_{B}L_{Bx}\nu_{yx-\mathrm{cell}}^{B}+\cdots \right)}^{2}$ could be taken as the objective function of the cell assembly, and condition 2 would be satisfied by adjusting the number of different cells in the HRVE row.

To obtain material geometries with extreme mechanical properties, two issues need to be addressed: i) the geometry of the cells corresponding to the extreme properties and ii) the spatial assembly layouts of these cells. According to the above analysis of the theoretical model, the extreme material properties require that the product of Poisson's ratios of the cells along the *x*‐ and *y*‐directions be as close to 1 as possible (condition 1: $1-\nu_{yx}^{m,n}\;\nu_{xy}^{m,n}=0$). Combined with the cumulative term in condition 2 (i.e., $\sum_{n=1}^{N}\delta_{n}\nu_{yx}^{m,n}=0$), the cells involved in the assembly should include a cell with PPR and a cell with NPR. In cell assembly, in addition to the same column arrangement and periodic arrangement, the row cell ratios that satisfy condition 2 are needed. Thus, design criteria are proposed accordingly, including a screening criterion for basic cell geometries with the required mechanical properties and an assembly criterion for specific spatial layouts of these cells (**Figure** **5**).

As the geometric architecture within the cell is not restricted, many structural design methods are available here for constructing cell geometries, such as the proposed classical architectures (used in this paper, see the “Design of HAMs” of Experimental Section for more details), initial architectures based on topology optimization, and artificially innovative architectures. In the screening criterion, an optimization model for the design of the cell structure was established by taking minimum: *f*_1_(***ρ*** ^***i***^)  = |1 − ν_*yx* − cell_ · ν_*xy* − cell_(***ρ*** ^***i***^)|  as the objective function to identify cell geometries with Poisson's ratio product closest to 1 (Figure S14, Supporting Information). Depending on the design method, the design variable ***ρ*** ^***i***^ can be either the relative density of the cell topology (for the topology optimization method) or the geometric parameters of the cell (for the geometric parameter optimization method). The screened cells were grouped according to whether they were of the same height *H* to ensure that different cells could be assembled into a continuous geometry. Because condition 2 is a cumulative term equal to zero, a second screening was performed to obtain cell groups containing both NPR and PPR cells (Figure 5). In this manner, cells within any screened group can be used as the basic cells for the assembly design and would be further assembled into an HRVE under the guidance of Condition 2.

In the assembly criterion, condition 2 was transformed into an objective function ($\mathrm{minimum}:\;f_{2}={\left(N_{A}L_{Ax}\nu_{yx-\mathrm{cell}}^{A}+N_{B}L_{Bx}\nu_{yx-\mathrm{cell}}^{B}+\cdots \right)}^{2}$) for obtaining the row cell ratio (e.g., *N_A_*:  *N_B_*). Because the material properties are independent of the arrangement sequence of the cells in the row (simulation results), the screened cells were assembled into an HRVE row based only on this number ratio. Having determined the row of the HRVE, the geometry of the HRVE and corresponding HAM were constructed according to the assembly methods shown in Figure 5 (i.e., the same column arrangement and periodic arrangement).

Furthermore, programmable material properties can be achieved by adjusting the number ratio of different cells in the HRVE rows (Figure S15, Supporting Information), whereas the property range is governed by the Poisson's ratio product of the basic cells (Figure S14, Supporting Information). With the premise of ensuring that the property requirements are satisfied, there is no specific requirement in the criteria for material distribution within the cells (e.g., topology, size, and shape). Therefore, these criteria are considered geometrically generic, that is, various cells with different geometries are available for this assembly design. (some reported materials were used to demonstrate the geometric generality of the criteria, see Figures S16 and S17 for more details, Supporting Information).

### 2.5 Mechanical Testing of HAMs

**Figure 6:**
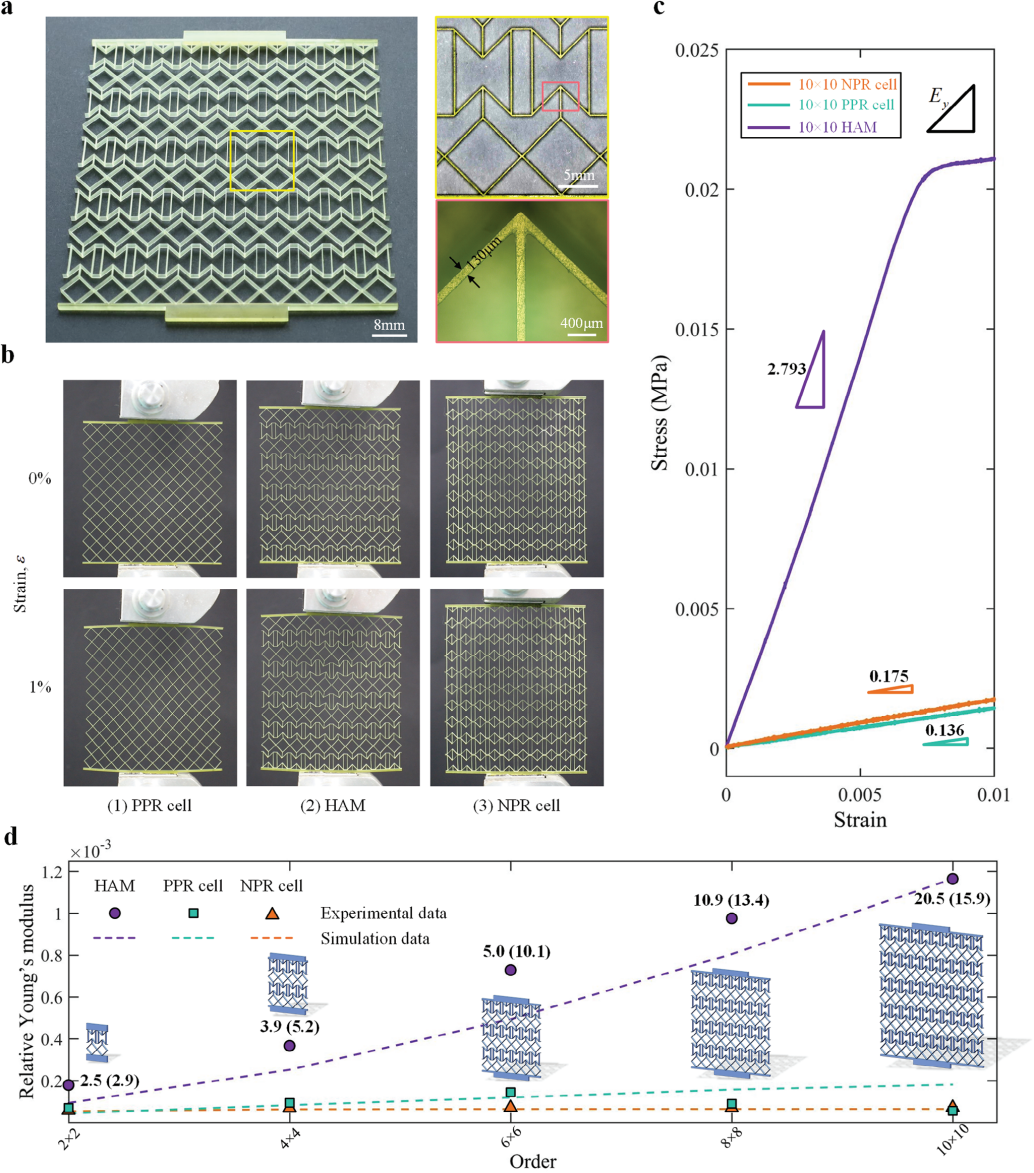
Experimental and numerical results. a) Images of 3D printed 10 × 10 HAM with geometry decomposition at each layer. Scale bars, 8 mm for HAM; 5 mm for HRVE; 400 µm for cell. b) Mechanical deformation response of 10 × 10 samples in uniaxial tensile testing (0% applied strain (top) and 1% applied strain (bottom)). c) Experimental stress–strain curves for all 10 × 10 samples (the measured data for other HAMs are shown in the Figure S18, Supporting Information). d) Experimental and simulation data of relative Young's modulus for materials at different assembly orders. The property enhancement multipliers of HAMs compared to homogeneous materials are shown in this figure (the values in parentheses are the enhancement multipliers of HAM with respect to the material formed by the NPR cell).

To verify the property enhancement effect of HAMs, we compared the mechanical responses of HAMs and homogeneous materials under uniaxial stretching. Considering the scale limitation of fabrication, the sequence of the design criteria was adjusted for the construction of HAMs with significantly enhanced Young's modulus (for more details, see the “Design of HAMs” of Experimental Section and Table S1, Supporting Information). Several material samples with different periodic assembly orders (i.e., from 2 × 2 to 10 × 10 order) were fabricated by adopting projection micro‐stereolithography 3D printing technique (the “Fabrication of HAMs” of Experimental Section) and stretched uniaxially using a vertical testing machine (see the “Mechanical testing” of Experimental Section for more details) (**Figure** **6**a,b).

Figure 6c shows the measured stress–strain curves for the 10 × 10 samples. As expected, the HAM exerted a significant enhancement effect on the Young's modulus (20.5‐fold enhancement compared to the material formed by the PPR cell and 15.9‐fold enhancement compared to the material formed by the NPR cell). Importantly, the evolution of the Young's modulus of HAMs at different assembly orders suggests that this enhancement effect can be further reinforced with increasing order (Figure 6d; Figure S19, Supporting Information). In fact, because of the attenuation of the boundary effect caused by the growing assembly order, the Young's modulus of HAMs shows a tendency to converge to the ideal properties of the material applied to PBCs, while the properties of the material formed by a single cell have converged to their respective such values (see Figure S19, Supporting Information).

### 2.6 HAMs with Extreme Young's Modulus

**Figure 7:**
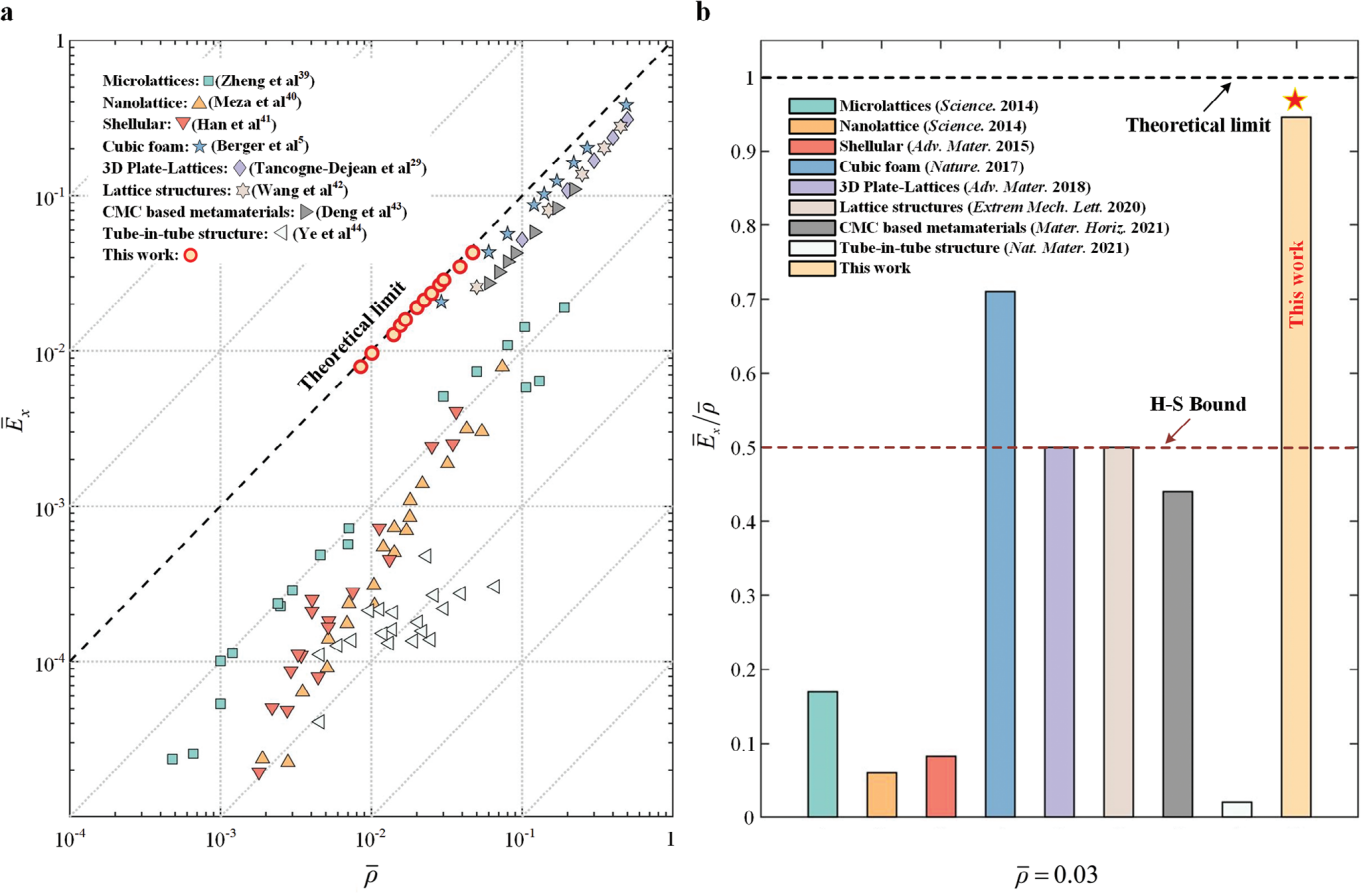
Young's modulus of HAMs and comparison with other materials. a) A comparison of Young's modulus between HAMs and other reported mechanical metamaterials.^[^ ^5^, ^29^, ^39^, ^40^, ^41^, ^42^, ^43^, ^44^ ^]^ The geometric parameters and elastic properties of these 12 HAMs with extreme Young's modulus are illustrated in the Table S2 (Supporting Information). b) Normalized Young's modulus ${\bar{E}}_{x}/\bar{\rho }$ of all materials at the relative density of $\bar{\rho }=0.03$ (Table S3, Supporting Information). The red and black dashed lines represent the Hashin–Shtrikman upper bounds (i.e., H‐S Bound) of Young's modulus for isotropic materials (${\bar{E}}_{x}/\bar{\rho }=0.5$) and the theoretical limit in Ashby plots, respectively.^[^ ^35^ ^]^

To demonstrate the full advantages of HAMs in mechanical properties, we designed HAMs with extreme Young's modulus according to the proposed criteria (see the “Design of HAMs” of Experimental Section for more details). Plots of relative Young's modulus ${\bar{E}}_{x}$ versus relative density $\bar{\rho }$ (**Figure** **7**a) were employed to evaluate elastic properties of these HAMs. Compared to reported metamaterials with excellent elastic stiffness,^[^ ^5^, ^29^, ^39^, ^40^, ^41^, ^42^, ^43^, ^44^ ^]^ HAMs exhibited a more extreme Young's modulus that approximated the theoretical limit in the Ashby plots (Table S2, Supporting Information).^[^ ^36^ ^]^ To clearly demonstrate the enormous advantage of HAMs in Young's modulus, the normalized Young's modulus ${\bar{E}}_{x}/\bar{\rho }$ of each material with relative density $\bar{\rho }=0.03$ is given (Figure 7b). Here, ${\bar{E}}_{x}/\bar{\rho }$ of HAM at this density (${\bar{E}}_{x}/\bar{\rho }=0.946$) far exceeded the Hashin–Shtrikman (H–S) upper bound for isotropic materials (${\bar{E}}_{x}/\bar{\rho }=0.5$),^[^ ^29^ ^]^ while it improved by 33% compared to the previous best‐performing cubic foam (Table S3, Supporting Information).^[^ ^5^ ^]^ Such extreme properties suggest that HAMs designed carefully can approach the Young's modulus of the constituent solid materials at very low relative densities. Furthermore, although 12 HAMs with different relative densities are given here, the design of HAMs with an extreme Young's modulus would be realized for any relative density because the criteria do not limit this value. More importantly, these HAMs assembled by cells with pre‐defined topologies indicate that the obtained properties are still constrained in this case. Thus, our design criteria provide an excellent platform for architected materials to exhibit more extreme properties.

### 2.7 Outlook

**Figure 8:**
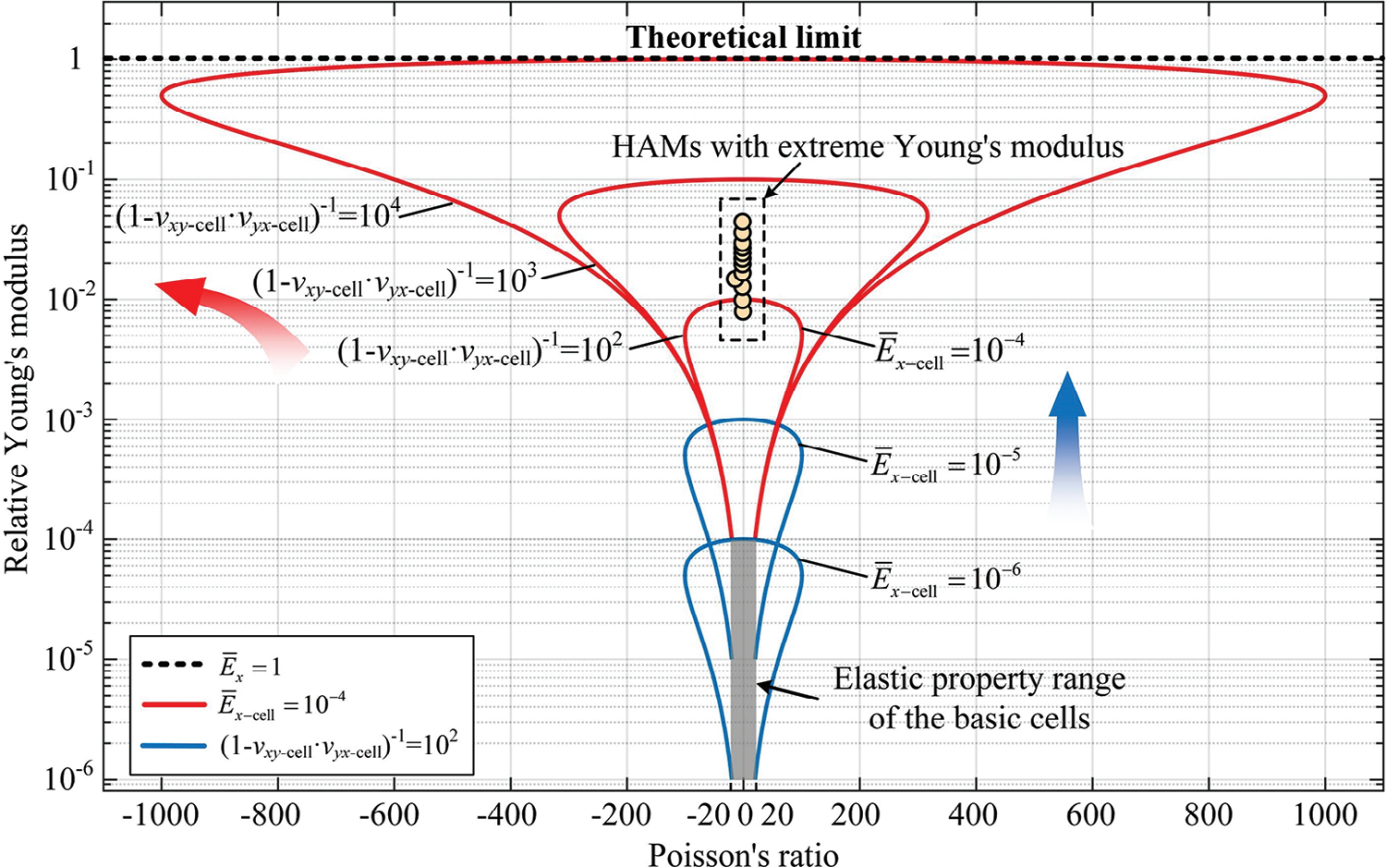
Elastic properties of HAMs assembled by cells with desirable mechanical properties. Evolution of the elastic properties for HAMs as a function of the corresponding properties and Poisson's ratio product for the cells. The figure provides one method to achieve the Young's modulus at the theoretical limit, that is, the relative Young's modulus of the cell is 10^−4^ while the cell Poisson's ratio product makes (1 − ν_*xy* − cell_ · ν_*yx* − cell_)^−1^ = 10^4^  holds. In this case, the Poisson's ratio of the material can be tuned arbitrarily in the range of (−1000, +1000). Meanwhile, the excellent Young's modulus and Poisson's ratio can be realized simultaneously.

Although the significant advantages of HAMs in terms of mechanical properties have been demonstrated, the property enhancement potential of such assembly design remains unknown. To explore this potential and determine the design space for the properties of HAMs, we further analyzed the relationship between the cell and material properties in our assembly strategy. By simplifying the theoretical model, a direct link between the cell and material properties was established (more detail can be found in ‘Section S2’, Supporting Information). As shown in **Figure** **8**, the material properties (i.e., ${\bar{E}}_{x}$) became more excellent with the increase of the corresponding properties of the cell (i.e., ${\bar{E}}_{x-\mathrm{cell}}$) when the cell Poisson's ratio product (i.e., ν_*xy* − cell_ · ν_*yx* − cell_) was determined (the blue line). With constant corresponding properties of the cells, the material properties were positively correlated with the degree to which the cell Poisson's ratio product approached 1 (the red line). Therefore, we can conclude that the upper limit of the mechanical properties of HAMs depends on both the corresponding properties and the Poisson's ratio product of the cells. More importantly, an unprecedented extreme design space of elastic properties would be achieved by assembling basic cells in the ordinary range (i.e., the gray area), including Young's modulus at the theoretical limit (i.e., ${\bar{E}}_{x}=1$ and shown by the black dashed line), tunable Poisson's ratios over an ultra‐large range (i.e., (−1000, +1000) in Figure 8, and this range would be further extended to the entire design space (−∞, +∞) when the mechanical properties of the cells are sufficiently desirable), and both superior Young's modulus and Poisson's ratio.

## 3 Conclusion

In this study, we proposed the generic criteria for the design of architected materials with programmable extreme mechanical properties. By applying these criteria to identify the cells with unique mechanical properties and their specific spatial layout, several material geometries with extreme Young's modulus were identified. Meanwhile, programmable properties adapted to various environments were easily realized by changing the number ratio of cells in the HRVE row (Figure S15, Supporting Information). Our criteria enabled cells with ordinary properties to exhibit extraordinary material properties through assembly, thereby providing the possibility of the design of materials capable of handling extreme tasks. Furthermore, the focus of the screening and assembly criteria is on the mechanical properties of the cells rather than on their topology, shape, and size (Figure S16, Supporting Information). Such features offer greater geometric design space and bring architected materials closer to real‐world applications.

In fact, the actual mechanical properties of HAMs are strongly associated with both their design and fabrication. As mentioned earlier, the upper limit of the material properties depends on the mechanical properties of the basic cells (Figure 8). Therefore, advanced design approaches are required to construct the cell geometry. Meanwhile, the developed fabrication techniques that make the actual material properties approximate the ideal extreme properties are also essential. Consequently, recent advances, including deep learning‐based optimization,^[^ ^45^ ^]^ data‐driven discovery in design approaches;^[^ ^23^ ^]^ large‐area projection micro‐stereolithography,^[^ ^39^ ^]^ and direct laser writing in fabrication techniques,^[^ ^46^ ^]^ offer exciting opportunities to achieve the proposed property enhancement potential. Thus, this research enables the design of materials to cover an unprecedented extreme range of properties.

## 4 Experimental Section

### Numerical Simulation

A finite element modeling (FEM) was used to simulate HAMs. To obtain the Young's modulus *E* and Poisson's ratio ν of materials, a linear elastic model in the plane stress state was considered in the commercial software ANSYS. Two boundary conditions were imposed on the model to simulate HAMs in the numerical experiments and tensile tests, respectively. Periodic boundary conditions (PBCs) allow the mimicking of HAMs with infinitely repeated arrangements using a single HRVE. Thus, this condition was applied to each HRVE model to evaluate the elastic properties of the HAMs in the numerical experiments. For the HAMs in the tensile tests, fixed constraints were imposed on all nodes at the upper and lower boundaries, while maintaining an upward uniform displacement at the top of the model. Quadrilateral elements (i.e., PLANE 182 with four nodes) were used to mesh of the HRVE geometries.

### Design of HAMs

The design of HAMs was divided into two parts: enhancement effect validation (mechanical testing) and extreme Young's modulus (simulation). To fulfill the assumptions of PBCs (i.e., an infinitely repeated arrangement) whenever possible, it is necessary to design HAMs containing more periodic assembly orders in the mechanical testing. Thus, considering the scale limitations of fabrication, the size of the HRVEs must be as small as possible. Here, the HRVE size depended on two factors: the cell size and cell number. Because the cell size was associated with the cell properties screened in the criteria, the cell number was prioritized for minimization. Specifically, according to the cell screening criterion (i.e., minimum: *f*_1_), the PPR cell was first identified by the geometric parameter optimization method using the 4‐STAR topology. In contrast to the criterion sequence, the row cell ratio corresponding to the maximum Young's modulus was preferentially set to 1:1 to minimize the cell number. Subsequently, by optimizing the geometric parameters of the honeycomb topology, the NPR cell was determined based on the cell assembly criterion (i.e., minimum:  *f*_2_). Finally, the order of the HRVE periodic assembly was identified based on the scale of fabrication.

To design HAMs with extreme Young's modulus, a geometric parameter optimization method was adopted to obtain the basic cells. Specifically, taking the honeycomb structure as the base topology, minimum:  *f*_1_ = |1 − ν_*yx* − cell_ · ν_*xy* − cell_|  as the objective function, the geometry of cells with NPR and PPR was constructed by optimizing key parameters including length (*L*, *C*, *l*, and *l*_1_), line width (*t*), and angle (θ) (Table S2, Supporting Information). The assembly principle for the same cell height was considered at this stage. Next, to assemble the cells into the HRVE, the row cell ratio corresponding to the maximum Young's modulus was determined by applying $\mathrm{minimum}:\;f_{2}={\left(N_{A}L_{Ax}\nu_{yx-\mathrm{cell}}^{A}+N_{B}L_{Bx}\nu_{yx-\mathrm{cell}}^{B}+\cdots \right)}^{2}$. Finally, the PBCs were applied to the HRVE to simulate HAMs with periodic arrangement patterns.

### Fabrication of HAMs

Each of the HAM specimens were fabricated with a BMF NanoArch S240 (precision of 5 µm) using projection micro‐stereolithography 3D printing technique. Photosensitive resin (Young's modulus of 2397 MPa) was used for the 3D printing of all specimens. The minimum size of samples was 130 µm. Before fabrication, the sample models were built using computer‐aided mapping software and converted into STL format for printing. These models were then sliced into a sequence of 2D images with pre‐defined layer thicknesses using slicing software. Next, the UV light shaped by the 2D image was irradiated onto the resin surface, bringing the material together uniformly toward the photopolymerization area. After structure printing of each layer was completed, the light continued to project the image of the next layer on top of the previous layer until the entire model was fabricated.

### Experiments

Uniaxial tensile testing of all specimens was performed on a vertical testing machine using a 50N load cell. In tensile testing, the top and bottom of the sample were fixed by the fixtures and the load was transmitted through these fixtures. The specimens were tensioned at ≈1% strain at an extension rate of 0.01 mms^−1^. The Young's modulus of the material was evaluated using the slope of the tensile stress–strain curve in the elastic stage.

### Statistical Analysis

Quantitative data points in Figure 6c,d are presented as mean ± standard deviation of at least three measurements. Statistical analyses were performed using MATLAB software (2020 version).

## Conflict of Interest

The authors declare no conflict of interest.

## Author Contributions

P.Y. and B.L. designed and conceived the research; P.Y. and Bang Li designed and carried out the numerical simulations; B.L. and H.L. analyzed the data; P.Y. and X.C. manufactured samples; H.J. and J.S. performed the experiments. B.L., J.H., X.C., and Y.L. supervised the research. All authors contributed to preparing and editing the manuscript.

## Supporting information

Supporting Information

Supplemental Movie 1

Supplemental Movie 2

Supplemental Movie 3

Supplemental Movie 4

## Data Availability

The data that support the findings of this study are available in the supplementary material of this article.
