# Supplementary material for: Design Criteria for Architected Materials with Programmable Mechanical Properties Within Theoretical Limit Ranges
Source: Adv Sci (Weinh). 2023 Dec 12;11(9):2307279. doi: 10.1002/advs.202307279 (PMC10916576; doi:10.1002/advs.202307279)
Supplement: Supplementary file 1 — Supporting Information [file ADVS-11-2307279-s004.pdf]

ADVANCED SCIENCE
Open Access

Supporting Information

for *Adv. Sci.*, DOI 10.1002/advs.202307279

Design Criteria for Architected Materials with Programmable Mechanical Properties Within Theoretical Limit Ranges

Peng Yin, Baotong Li, Jun Hong*, Hui Jing, Bang Li, Honglei Liu, Xiaoming Chen*, Yang Lu and Jinyou Shao*

Supporting Information

Design criteria for architected materials with programmable mechanical properties within theoretical limit ranges

Authors**:** Peng Yin, Baotong Li, Jun Hong, Hui Jing, Bang Li, Honglei Liu, Xiaoming Chen, Yang Lu, Jinyou Shao

This file includes:

Supplementary Section 1: Elastic properties of HAMs (including from S1.1 to S1.3)
Supplementary Section 2: The property enhancement potential
Figure S1 to S19 partially with explanatory text
Table S1
Table S2
Table S3
Legends for 4 Supplementary Movies

Section 1: Elastic properties of HAMs

Here, we propose a theoretical model to guide the determination of the basic cells and spatial layout in HAMs with extreme elastic properties. Inspired by the simulation results (i.e., HAMs with extreme properties have the same cell geometry in each column), our model focuses on handling the property prediction of HAMs with the same column arrangement. Specifically, the HAMs are periodically assembled by HRVEs with *M* rows and *N* columns, while the HRVEs are composed of rectangular region cells connected by boundary points (red dots refer to row connections and blue dots refer to column connections) (**Figure S1**). When the material is subjected to a uniaxial tensile stress σ_x along the *x*-direction, the Young's modulus of the material is

$$E_x = \frac{\sigma_x}{\varepsilon_x} \tag{S1}$$

where ε_x is the material strain in the *x*-direction. The Young's modulus E_x is normalized by the Young's modulus of the constituent material E_s, referred to as the relative Young's modulus $\overline{E}_x$, and is given by

$$\overline{E}_x = \frac{E_x}{E_s} \tag{S2}$$

and the Poisson's ratio of the material is

$$v_{xy} = -\frac{\varepsilon_y}{\varepsilon_x} \tag{S3}$$

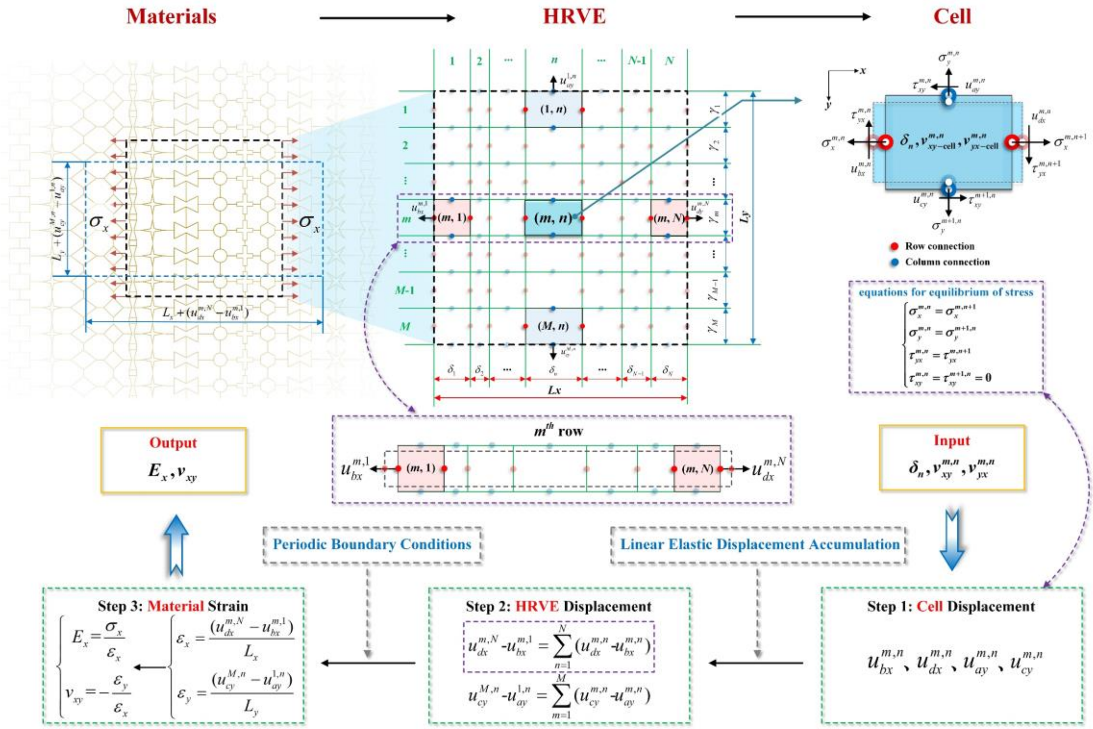

Figure S1. A theoretical model of HAMs for elastic property prediction.

According to the definition of material properties, there are three key parameters that need to be solved for the prediction of the elastic properties: the material strain, the HRVE displacement, and the cell displacement (Figure S1).

S1.1 Cell displacement

Assuming that the theoretical model is a linear elastic model, the cell displacement is defined as the displacement difference of the connection points on the parallel boundary of the cell. In this linear elastic model, the displacement field of the cell in *x* and *y* directions can be assumed to be a quadratic function of *x* and *y*.

$$u_x^{m,n}(x,y) = a_1 + a_2 x + a_3 y + a_4 x^2 + a_5 xy + a_6 y^2 \tag{S4}$$

$$u_y^{m,n}(x,y) = b_1 + b_2 x + b_3 y + b_4 x^2 + b_5 xy + b_6 y^2 \tag{S5}$$

The strain field of the cell can be expressed in terms of the displacement field as

$$\varepsilon_x^{m,n}(x,y) = \frac{\partial u_x^{m,n}}{\partial x} \tag{S6}$$

$$\varepsilon_y^{m,n}(x,y) = \frac{\partial u_y^{m,n}}{\partial y} \tag{S7}$$

$$\gamma_{xy}^{m,n}(x,y) = \frac{\partial u_x^{m,n}}{\partial y} + \frac{\partial u_y^{m,n}}{\partial x} \tag{S8}$$

The stress-strain relationship is strongly associated with the isotropy and anisotropy of the structural mechanical properties. For axisymmetric or rotationally symmetric geometries, the elastic properties could be assumed as isotropy along two principal axes (i.e., the elastic constants in the *x*- and *y*- directions are equal).[1] However, for other geometries, the elastic constants in different directions should be distinguished. Thus, the strain field of the cell with arbitrary geometry is given by

$$\begin{Bmatrix} \varepsilon_x \\ \varepsilon_y \\ \gamma_{xy} \end{Bmatrix} = \begin{bmatrix} 1/E_x & -\nu_{yx}/E_y & 0 \\ -\nu_{xy}/E_x & 1/E_y & 0 \\ 0 & 0 & 1/G_{xy} \end{bmatrix} \begin{Bmatrix} \sigma_x \\ \sigma_y \\ \tau_{xy} \end{Bmatrix} \tag{S9}$$

where the cell strain field consisting of the *x*-direction normal strain ε_x, the *y*-direction normal strain ε_y and the *x*-direction shear strain γ_{xy} is correlated with the cell stress field consisting of the *x*-direction normal stress σ_x, the *y*-direction normal stress σ_y and the *x*-direction shear stress τ_{xy} by the elastic matrix. The components of the elastic constants in different directions are given in this elastic matrix, e.g., the Young's modulus *E* has components E_x and E_y in the *x*- and *y*-directions, as do the Poisson's ratio *v* and the bulk modulus *G*.

Because the cell stress in the linear elastic range varies linearly, the stress distribution of the (*m*, *n*) cell is given by

$$\sigma_x^{m,n}(x,y) = \frac{1}{L_{nx}}\left[\sigma_x^{m,n+1}x + \sigma_x^{m,n}(L_{nx}-x)\right] + \frac{1}{L_{my}}(L_{my}-2y)\left(\tau_{yx}^{m,n+1}-\tau_{yx}^{m,n}\right) \tag{S10}$$

$$\sigma_y^{m,n}(x,y) = \frac{1}{L_{my}}\left[\sigma_y^{m,n+1}y + \sigma_y^{m,n}(L_{my}-y)\right] + \frac{1}{L_{nx}}(L_{nx}-2x)\left(\tau_{xy}^{m+1,n}-\tau_{xy}^{m,n}\right) \tag{S11}$$

$$\tau_{xy}^{m,n}(x,y) = \frac{1}{L_{nx}}\left[\tau_{yx}^{m,n+1}x + \tau_{yx}^{m,n}(L_{nx}-x)\right] + \frac{1}{L_{my}}\left[\tau_{xy}^{m+1,n}y + \tau_{xy}^{m,n}(L_{my}-y)\right] \tag{S12}$$

where $\sigma_x^{m,n}$ is the *x*-direction normal stress at the vertical interface between cell (*m*, *n*) and cell (*m*, *n*-1), and other cell stresses at connections are shown in Figure S1. L_{my} and L_{nx} are the width of the *m*-th row in the *y*-direction and the length of the *n*-th column in the *x*-direction, respectively.

To ensure that the periodic array of HRVEs represents a continuous physical body, two continuities need to be satisfied at the boundaries of adjacent HRVEs: (i) the displacements must be continuous, i.e., adjacent HRVEs cannot be separated or encroach into each other at the deformed boundaries, which means that the displacement differences at corresponding positions on the parallel boundaries are equal; (ii) the forces at the parallel boundaries must be the same.[2] Meanwhile, in the case of the same column arrangement, an HRVE with *M* rows and *N* columns of cells is equivalent to an HRVE with 1 row and *N* columns of cells due to the PBCs. As a result, the stresses of the connection points on the upper and lower boundaries are the same for any cells on such HRVE row, and there is no tangential stress (**Figure S2**). The stress equilibrium equation on the cell boundary can thus be expressed as

$$
\begin{cases}
\sigma_x^{m,n} = \sigma_x^{m,n+1} \\
\sigma_y^{m,n} = \sigma_y^{m+1,n} \\
\tau_{yx}^{m,n} = \tau_{yx}^{m,n+1} \\
\tau_{xy}^{m,n} = \tau_{xy}^{m+1,n} = 0
\end{cases}
\tag{S13}
$$

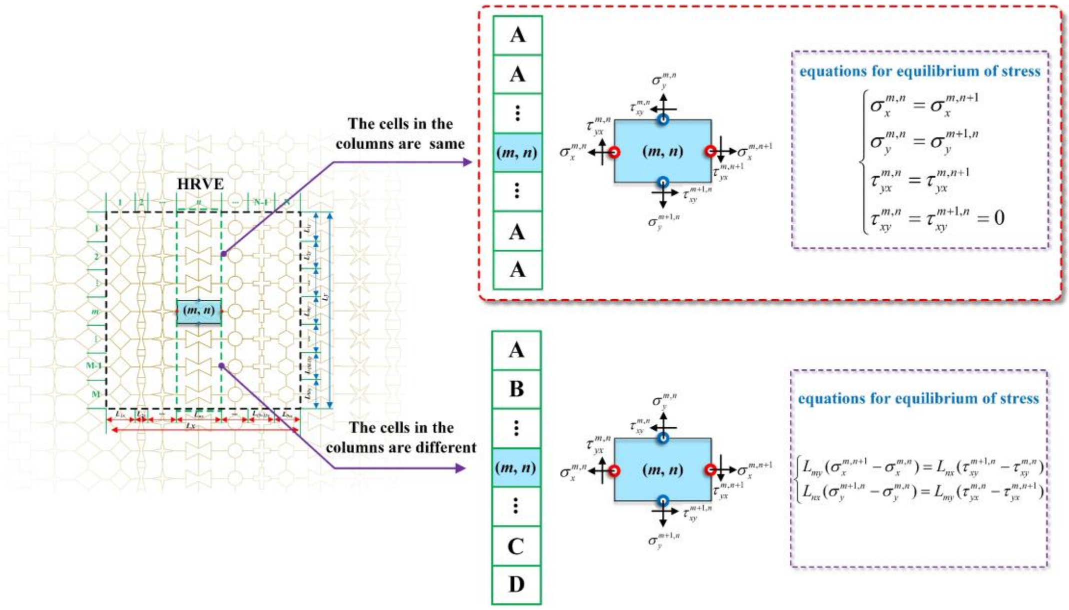

Figure S2. The local stress equilibrium of cells in the same column arrangement.

Substituting Equation S13 into Equation S10, S11 and S12, the stress field of the (*m*, *n*) cell in the same column arrangement can be written as

$$\sigma_x^{m,n}(x,y) = \sigma_x^{m,n} \tag{S14}$$

$$\sigma_y^{m,n}(x,y) = \sigma_y^{m,n} \tag{S15}$$

$$\tau_{xy}^{m,n}(x,y) = \tau_{yx}^{m,n} \tag{S16}$$

Next, the cell displacement field could be calculated by solving the unknown coefficients in Equation S4 and S5. To determine these coefficients, different expressions of the same displacement field are given by simultaneously completing the substitution of Equation S14, S15 and S16 into Equation S9 and the substitution of Equation S4 and S5 into Equation S6, S7 and S8. Thus, these coefficients can be expressed as

$$a_2 = \frac{1}{E_x^{m,n}}\sigma_x^{m,n} - \frac{v_{yx}^{m,n}}{E_y^{m,n}}\sigma_y^{m,n} \tag{S17}$$

$$b_3 = \frac{1}{E_y^{m,n}}\sigma_y^{m,n} - \frac{v_{xy}^{m,n}}{E_x^{m,n}}\sigma_x^{m,n} \tag{S18}$$

$$a_3 + b_2 = \frac{1}{G_{xy}}\tau_{yx}^{m,n} \tag{S19}$$

$$a_4 = a_5 = a_6 = b_4 = b_5 = b_6 = 0 \tag{S20}$$

Since the tangential stress in the y-direction $\tau_{yx}^{m,n}$ does not affect the displacement in the x-direction, the coefficient $a_3 = 0$ and $b_2 = \tau_{yx}^{m,n} / G_{xy}$. Thus, Equation S4 and S5 can be rewritten as

$$u_x^{m,n}(x,y) = a_1 + \left(\frac{1}{E_x^{m,n}} \sigma_x^{m,n} - \frac{\nu_{yx}^{m,n}}{E_y^{m,n}} \sigma_y^{m,n} \right) x \tag{S21}$$

$$u_y^{m,n}(x,y) = b_1 + \left(\frac{1}{E_y^{m,n}} \sigma_y^{m,n} - \frac{\nu_{xy}^{m,n}}{E_x^{m,n}} \sigma_x^{m,n} \right) y + \frac{1}{G_{xy}} \tau_{yx}^{m,n} x \tag{S22}$$

Note that a_1 and b_1 would be determined by substituting the displacement of any point in the cell, which does not influence the relative displacement of connection points on the cell parallel boundary.

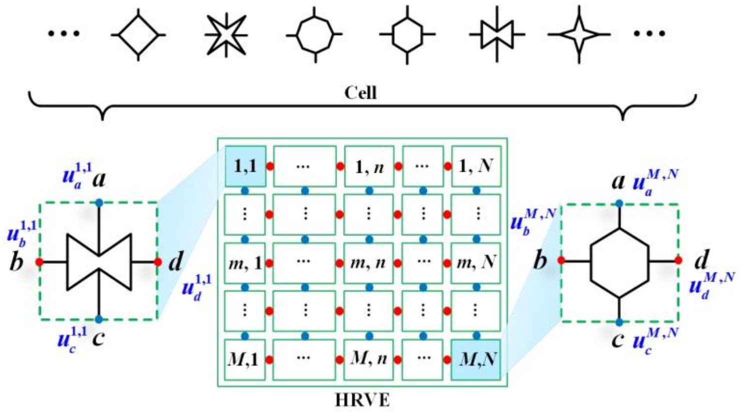

Figure S3. Illustration of cell displacement.

As shown in **Figure S3**, the cell displacements in x-direction could be determined by the displacement difference of the row connection points (red dots) on the parallel boundary. Similarly, the cell displacements in y-direction could be determined by the displacement difference of the column connection points (blue dots) on the parallel boundary. For example, $u_{dx}^{M,N} - u_{bx}^{M,N}$ and $u_{cy}^{M,N} - u_{ay}^{M,N}$ denote the displacement of the (M, N) cell in the x- and y- direction, respectively. Therefore, by substituting the coordinates of connection points, the (m, n) cell displacement in the x- and y-directions is expressed as

$$u_{dx}^{m,n} - u_{bx}^{m,n} = L_{nx} \left(\frac{1}{E_x^{m,n}} \sigma_x^{m,n} - \frac{\nu_{yx}^{m,n}}{E_y^{m,n}} \sigma_y^{m,n} \right) \tag{S23}$$

$$u_{cy}^{m,n} - u_{ay}^{m,n} = L_{my} \left(\frac{1}{E_y^{m,n}} \sigma_y^{m,n} - \frac{\nu_{xy}^{m,n}}{E_x^{m,n}} \sigma_x^{m,n} \right) \tag{S24}$$

S1.2 HRVE displacement and periodic boundary conditions (PBCs)

Here, the HRVE displacement is defined as the displacement difference of connection points at the corresponding position on the parallel boundary of the HRVE. Considering the linear elastic model, a linear accumulation is adopted in the construction of the relation between the cell displacement and the HRVE displacement.

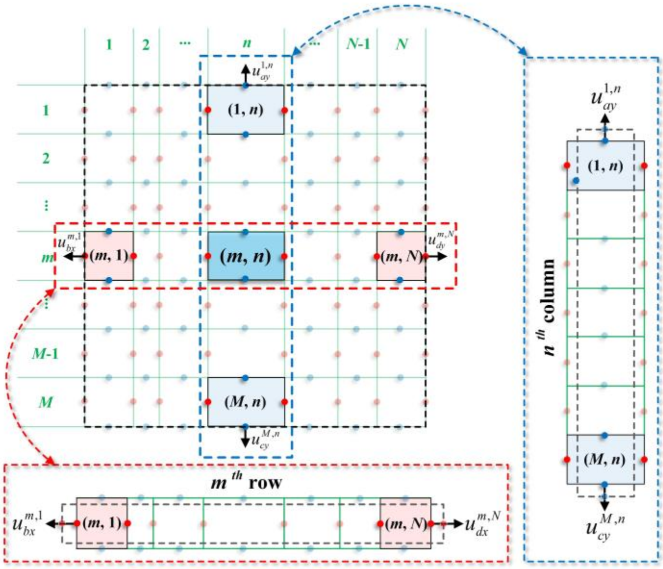

Figure S4. Illustration of HRVE displacement.

In **Figure S4**, the HRVE displacement of the *m*-th row in the *x*-direction can be obtained by accumulating the *x*-direction displacements of all cells on that row (red dashed box). Similarly, the HRVE displacement of the *n*-th column in the *y*-direction can be obtained by accumulating the *y*-direction displacements of all cells on that column (blue dashed box). Thus, the displacement of the HRVE with *M* rows and *N* columns are given by

$$u_{dx}^{m,N} - u_{bx}^{m,1} = \sum_{n=1}^{N} (u_{dx}^{m,n} - u_{bx}^{m,n}) \tag{S25}$$

$$u_{cy}^{M,n} - u_{ay}^{1,n} = \sum_{m=1}^{M} (u_{cy}^{m,n} - u_{ay}^{m,n}) \tag{S26}$$

where $u_{dx}^{m,N} - u_{bx}^{m,1}$ is the HRVE displacement of the *m*-th row in the *x*- direction, $u_{cy}^{M,n} - u_{ay}^{1,n}$ is the HRVE displacement of the *n*-th column in the *y*- direction. Substituting Equation S23 and S24 into Equation S25 and S26, yielding

$$u_{dx}^{m,N} - u_{bx}^{m,1} = \sum_{n=1}^{N} L_{nx} \left(\frac{1}{E_x^{m,n}} \sigma_x^{m,n} - \frac{v_{yx}^{m,n}}{E_y^{m,n}} \sigma_y^{m,n} \right) \tag{S27}$$

$$u_{cy}^{M,n} - u_{ay}^{1,n} = \sum_{m=1}^{M} L_{my} \left(\frac{1}{E_y^{m,n}} \sigma_y^{m,n} - \frac{v_{xy}^{m,n}}{E_x^{m,n}} \sigma_x^{m,n} \right) \tag{S28}$$

The periodic boundary condition (PBC) is a numerical method for calculating the elastic properties of materials with periodic arrangement characteristics.[2] As shown in Figure S1, because the displacements of the points at corresponding positions on the HRVE parallel boundary are the same (displacement continuity constraint of PBC), the HRVE displacements in the *x*-direction of each row are equal and can be expressed as

$$u_{dx}^{m,N} - u_{bx}^{m,1} = u_{dx}^{1,N} - u_{bx}^{1,1} = L_x \varepsilon_x \tag{S29}$$

Similarly, the HRVE displacements in the *y*-direction of each column are also equal and written as

$$u_{cy}^{M,n} - u_{ay}^{1,n} = u_{cy}^{M,1} - u_{ay}^{1,1} = L_y \varepsilon_y \tag{S30}$$

where L_x and L_y denote the length of HRVE in the *x*- and *y*- directions, respectively.

Besides the HRVE displacement, the local stresses of connection points on the cell parallel boundary are also affected by PBC. Under the unidirectional load in *x*-direction, the local stress equilibrium in the first row and the first column of the HRVE is

$$\frac{1}{L_y} \sum_{m=1}^{M} L_{my} \sigma_x^{m,1} = \sigma_x \tag{S31}$$

$$\frac{1}{L_x} \sum_{n=1}^{N} L_{nx} \sigma_y^{1,n} = 0 \tag{S32}$$

and the geometric length ratio of the cell in the HRVE is expressed as

$$\eta_m = \frac{L_{my}}{L_y} \tag{S33}$$

$$\delta_n = \frac{L_{nx}}{L_x} \tag{S34}$$

in which η_m and δ_n denote the length ratio of the cell in the *m*-th row to the whole HRVE in the *y*-direction and the length ratio of the cell in the *n*-th column to the whole HRVE in the *x*-direction, respectively. Substitution of Equation S33 and S34 into Equation. S31 and S32 yields

$$\sum_{m=1}^{M} \eta_m \sigma_x^{m,1} = \sigma_x \tag{S35}$$

$$\sum_{n=1}^{N} \delta_n \sigma_y^{1,n} = 0 \tag{S36}$$

Based on the stress equilibrium on the cell boundary in Equation S13, the local normal stress equilibrium of any column in the *x*-direction is

$$\sum_{m=1}^{M} \eta_m \sigma_x^{m,n} = \sum_{m=1}^{M} \eta_m \sigma_x^{m,1} = \sigma_x \tag{S37}$$

Similarly, for any row, the local normal stress equilibrium in the *y*-direction can be expressed as

$$\sum_{n=1}^{N} \delta_n \sigma_y^{m,n} = \sum_{n=1}^{N} \delta_n \sigma_y^{1,n} = 0 \tag{S38}$$

S1.3 Material strain and elastic property

According to the definitions of Young's modulus and Poisson's ratio in Equation S1 and S3, it is critical to solve the material strains in the *x*- and *y*- directions. The relationship between material strain and HRVE displacement has been given in Equation S29 and S30. Considering the HRVE displacement of *m*-th row in the *x*-direction, and substituting Equation S27 and S34 into Equation S29, we can get the material strain in the *x*-direction.

$$\varepsilon_x = \sum_{n=1}^{N} \delta_n \left(\frac{1}{E_x^{m,n}} \sigma_x^{m,n} - \frac{v_{yx}^{m,n}}{E_y^{m,n}} \sigma_y^{m,n} \right) \tag{S39}$$

Similarly, by substituting Equation S28 and S33 into Equation S30, the material strain in the *y*-direction is

$$\varepsilon_y = \sum_{m=1}^{M} \eta_m \left(\frac{1}{E_y^{m,n}} \sigma_y^{m,n} - \frac{v_{xy}^{m,n}}{E_x^{m,n}} \sigma_x^{m,n} \right) \tag{S40}$$

In case of the same column arrangement, Equation S40 can be expressed as

$$E_y^{m,n} \varepsilon_y = \sum_{m=1}^{M} \eta_m \sigma_y^{m,n} - \frac{v_{xy}^{m,n} E_y^{m,n}}{E_x^{m,n}} \sum_{m=1}^{M} \eta_m \sigma_x^{m,n} \tag{S41}$$

Next, Equation S41 is multiplied by δ_n simultaneously and accumulates all the columns in HRVEs. Due to $E_x v_{yx} = E_y v_{xy}$ (symmetry of the constitutive matrix) and the invariance of the material strain ε_y of each column in the HRVE (displacement continuity constraint of PBC), we can get

$$\sum_{n=1}^{N} \delta_n E_y^{m,n} \varepsilon_y = \sum_{m=1}^{M} \eta_m \sum_{n=1}^{N} \delta_n \sigma_y^{m,n} - \sum_{n=1}^{N} \delta_n v_{yx}^{m,n} \sum_{m=1}^{M} \eta_m \sigma_x^{m.n} \tag{S42}$$

Substituting Equation S37 and S38 into Equation S42, the material strain ε_y in the *y*-direction can rewritten as

$$\varepsilon_y = -\left[\left(\sum_{n=1}^{N} \delta_n v_{yx}^{m,n} \right) / \left(\sum_{n=1}^{N} \delta_n E_y^{m,n} \right) \right] \sigma_x \tag{S43}$$

The unknown local stress has been eliminated here.

By multiplying the Equation S39 by η_m simultaneously and accumulating all the rows in HRVEs, we obtain

$$\sum_{m=1}^{M} \eta_m \varepsilon_x = \sum_{n=1}^{N} \delta_n \left[\frac{1}{E_x^{m,n}} \sum_{m=1}^{M} \eta_m \sigma_x^{m,n} - \frac{v_{yx}^{m,n}}{E_y^{m,n}} \sum_{m=1}^{M} \eta_m \sigma_y^{m,n} \right] \tag{S44}$$

and

$$\varepsilon_x = \sum_{n=1}^{N} \frac{\delta_n}{E_x^{m,n}} \sum_{m=1}^{M} \eta_m \sigma_x^{m,n} - \sum_{n=1}^{N} \frac{\delta_n v_{yx}^{m,n}}{E_y^{m,n}} \sum_{m=1}^{M} \eta_m \sigma_y^{m,n} \tag{S45}$$

According to Equation S41, $\sum_{m=1}^{M} \eta_m \sigma_y^{m,n}$ can be expressed as

$$\sum_{m=1}^{M} \eta_m \sigma_y^{m,n} = E_y^{m,n} \varepsilon_y + \frac{v_{xy}^{m,n} E_y^{m,n}}{E_x^{m,n}} \sum_{m=1}^{M} \eta_m \sigma_x^{m,n} \tag{S46}$$

Substituting Equation S37 and S46 into Equation S45, yielding

$$\varepsilon_x = \sum_{n=1}^{N} \frac{\delta_n}{E_x^{m,n}} \sigma_x - \sum_{n=1}^{N} \frac{\delta_n \nu_{yx}^{m,n}}{E_y^{m,n}} \left(E_y^{m,n} \varepsilon_y + \frac{\nu_{xy}^{m,n} E_y^{m,n}}{E_x^{m,n}} \sigma_x \right) \tag{S47}$$

Further, substituting Equation S43 into Equation S47, the material strain in the *x*-direction is rewritten as

$$\varepsilon_x = \left[\sum_{n=1}^{N} \frac{\delta_n \left(1 - \nu_{xy}^{m,n} \nu_{yx}^{m,n}\right)}{E_x^{m,n}} + \left(\sum_{n=1}^{N} \delta_n \nu_{yx}^{m,n} \right)^2 / \left(\sum_{n=1}^{N} \delta_n E_y^{m,n} \right) \right] \sigma_x \tag{S48}$$

Thus, the Young's modulus of HAMs can be expressed as

$$E_x = \frac{\sigma_x}{\varepsilon_x} = \frac{1}{\sum_{n=1}^{N} \frac{\delta_n \left(1 - \nu_{xy}^{m,n} \nu_{yx}^{m,n}\right)}{E_x^{m,n}} + \left(\sum_{n=1}^{N} \delta_n \nu_{yx}^{m,n} \right)^2 / \left(\sum_{n=1}^{N} \delta_n E_y^{m,n} \right)} \tag{S49}$$

and the relative Young's modulus is

$$\bar{E}_x = \frac{E_x}{E_s} = \frac{1}{\sum_{n=1}^{N} \frac{\delta_n \left(1 - \nu_{xy}^{m,n} \nu_{yx}^{m,n}\right)}{\bar{E}_x^{m,n}} + \left(\sum_{n=1}^{N} \delta_n \nu_{yx}^{m,n} \right)^2 / \left(\sum_{n=1}^{N} \delta_n \bar{E}_y^{m,n} \right)} \tag{S50}$$

Substituting Equation S43 and S48 into Equation S3, the Poisson's ratio of HAMs can be written as

$$\nu_{xy} = -\frac{\varepsilon_y}{\varepsilon_x} = \frac{1}{\frac{\left(\sum_{n=1}^{N} \delta_n \frac{1 - \nu_{yx}^{m,n} \nu_{xy}^{m,n}}{\bar{E}_x^{m,n}} \right) \sum_{n=1}^{N} \delta_n \bar{E}_y^{m,n}}{\sum_{n=1}^{N} \delta_n \nu_{yx}^{m,n}} + \sum_{n=1}^{N} \delta_n \nu_{yx}^{m,n}} \tag{S51}$$

Section 2: The property enhancement potential

To explore the property enhancement potential of the assembly design, a developed theory is presented based on the above theoretical model. To reduce the adverse effects caused by too many cell types, we start by taking a set of positive and negative Poisson's ratio cell pairs (i.e., cell *A* and cell *B*). Thus, Equation S50 and S51 can be rewritten as

$$\bar{E}_x = \frac{1}{\frac{N_A \delta_A (1 - \nu_{xy}^A \nu_{yx}^A)}{\bar{E}_x^A} + \frac{N_B \delta_B (1 - \nu_{xy}^B \nu_{yx}^B)}{\bar{E}_x^B} + \frac{(N_A \delta_A \nu_{yx}^A + N_B \delta_B \nu_{yx}^B)^2}{N_A \delta_A \bar{E}_y^A + N_B \delta_B \bar{E}_y^B}} \tag{S52}$$

$$\nu_{xy} = \frac{1}{\frac{\left(\frac{N_A \delta_A (1 - \nu_{xy}^A \nu_{yx}^A)}{\bar{E}_x^A} + \frac{N_B \delta_B (1 - \nu_{xy}^B \nu_{yx}^B)}{\bar{E}_x^B} \right) \left(N_A \delta_A \bar{E}_y^A + N_B \delta_B \bar{E}_y^B \right)}{N_A \delta_A \nu_{yx}^A + N_B \delta_B \nu_{yx}^B} + \left(N_A \delta_A \nu_{yx}^A + N_B \delta_B \nu_{yx}^B \right)} \tag{S53}$$

where N_A and N_B are the number of cell *A* and cell *B* in the HRVE row, respectively. To more clearly characterize the relationship between the elastic properties of HAMs and basic cells, the

following simplifying assumptions about the mechanical properties of the cells are made here.

$$\begin{cases} \overline{E}_x^A = \overline{E}_x^B = \overline{E}_{x-\text{cell}} \\ \overline{E}_y^A = \overline{E}_y^B \\ \left|v_{xy}^A\right| = \left|v_{xy}^B\right| = v_{xy-\text{cell}} \\ v_{xy}^A = -v_{xy}^B \\ v_{yx}^A = -v_{yx}^B \\ v_{xy}^A v_{yx}^A = v_{xy}^B v_{yx}^B = v_{xy-\text{cell}} v_{yx-\text{cell}} \end{cases} \tag{S54}$$

where $\overline{E}_{x-\text{cell}}$ is the relative Young's modulus of the basic cells. $v_{xy-\text{cell}}$ and $v_{yx-\text{cell}}$ are the Poisson's ratios of the basic cells in the *x*- and *y*-directions, respectively. These simplifying assumptions can be achieved through some advanced structural design methods.[3] Substituting Equation S54 into Equation S52 and S53, yielding

$$\overline{E}_x = \frac{\overline{E}_{x-\text{cell}}}{(1-v_{xy-\text{cell}}v_{yx-\text{cell}}) + v_{xy-\text{cell}}v_{yx-\text{cell}}(f_A - f_B)^2} \tag{S55}$$

$$v_{xy} = \frac{v_{xy-\text{cell}}}{\dfrac{1-v_{xy-\text{cell}}v_{yx-\text{cell}}}{(f_A - f_B)} + v_{xy-\text{cell}}v_{yx-\text{cell}}(f_A - f_B)} \tag{S56}$$

in which

$$\begin{cases} f_A = N_A\delta_A \\ f_B = N_B\delta_B \end{cases} \tag{S57}$$

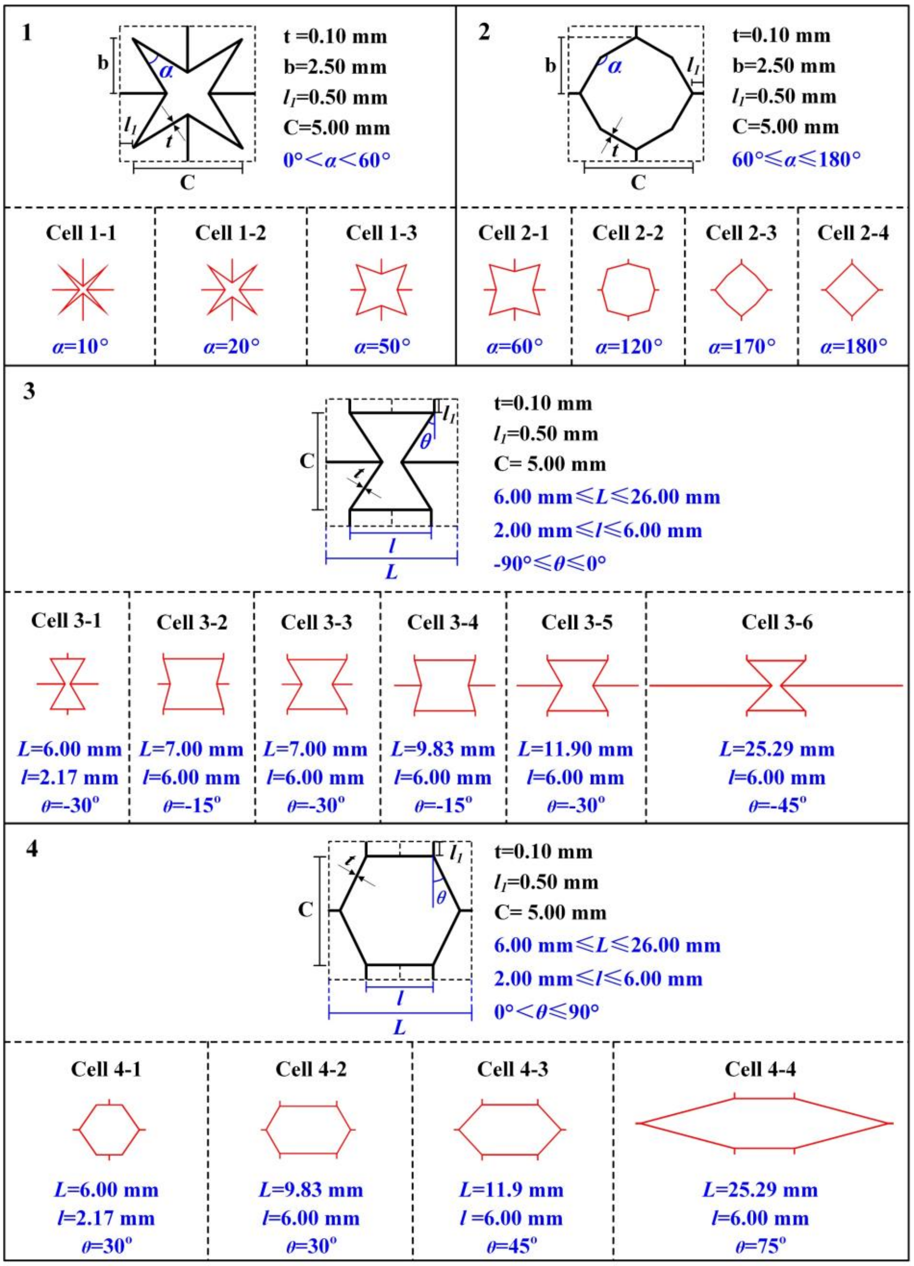

Figure S5. Geometric details of basic cells in the numerical experiments. To construct the basic cell for heterogeneous assembly in the numerical experiments, two classical architectures, including 4-star system[4] (isotropy) and honeycomb[5](anisotropy), are first chosen. According to the positive or negative of Poisson's ratio, 4 topologies (i.e., black structures, from 1 to 4 in the figure) are then derived from the classical architectures. By changing the geometric parameters (i.e., length, L and l, angle, θ and α) of these topologies, 17 basic cell geometries (i.e., red structures, from Cell 1-1 to Cell 4-4) are finally constructed. To ensure the cells to assemble into a continuous geometry, the height of all cells is the same.

Cell	Elastic properties	Anisotropy/ Isotropy	PPR/ NPR
**Cell 1-1**	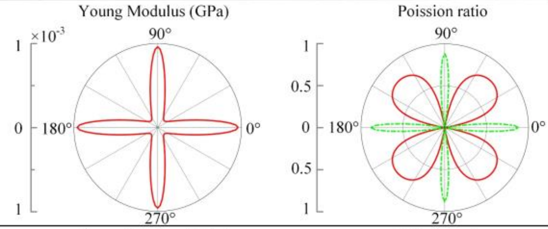	**Isotropy**	**NPR**
**Cell 1-2**	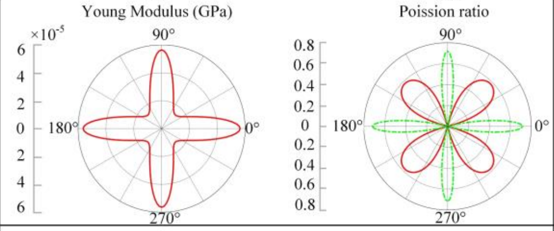	**Isotropy**	**NPR**
**Cell 1-3**	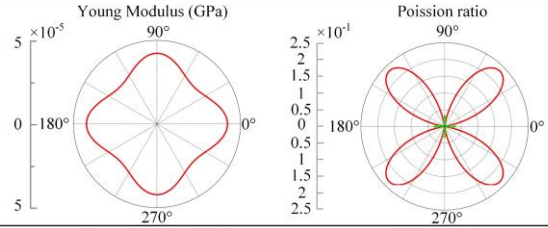	**Isotropy**	**PPR**
**Cell 2-1**	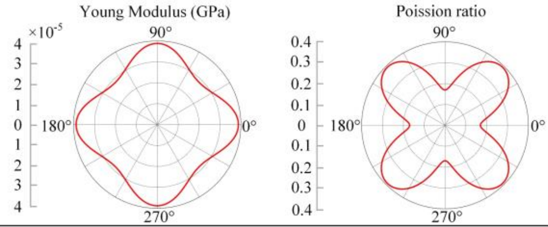	**Isotropy**	**NPR**
**Cell 2-2**	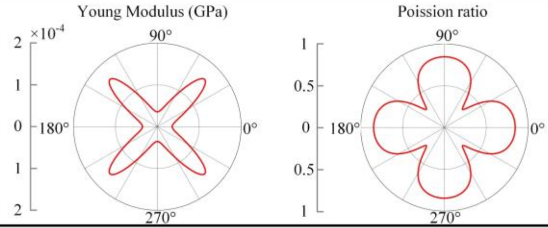	**Isotropy**	**PPR**

Figure S6. Elastic properties of basic cells in the Numerical Experiments. Young's modulus curves and Poisson's ratio curves of 17 basic cells (this part includes Cell 1-1 to Cell 2-2). These cells exhibit great diversity in elastic properties, i.e., Young's modulus from isotropy to anisotropy, and Poisson's ratios from positive (PPR) to negative (NPR). Further, all combinations of these elastic properties are covered, such as, isotropy + NPR (from Cell 1-1 to Cell 1-3), isotropy + PPR (from Cell 2-1 to Cell 2-4), anisotropy + NPR (from Cell 3-1 to Cell 3-6), and anisotropy + PPR (from Cell 4-1 to Cell 4-4). Note that difference colors in the Poisson's ratio curves are used to distinguish the positive and negative of Poisson's ratio, i.e., red denotes PPR and green denotes NPR. The simultaneous presence of these two colors indicates that the same cell might exhibit positive and negative opposite Poisson's ratios in different directions.

Cell	Elastic properties	Anisotropy/ Isotropy	PPR/ NPR
**Cell 2-3**	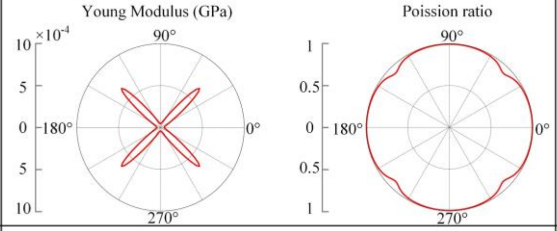	**Isotropy**	**PPR**
**Cell 2-4**	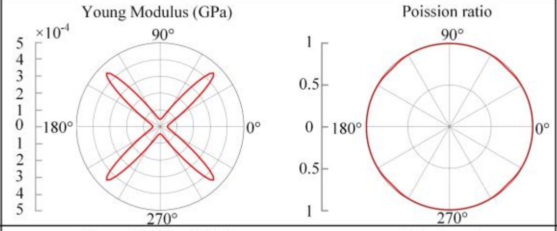	**Isotropy**	**PPR**
**Cell 3-1**	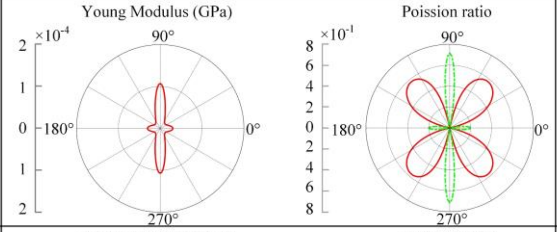	**Anisotropy**	**NPR**
**Cell 3-2**	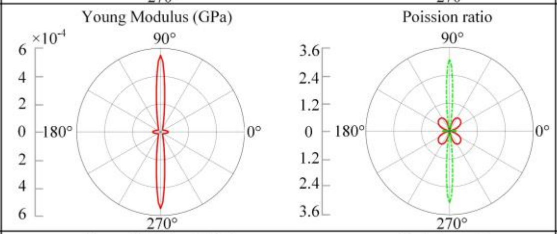	**Anisotropy**	**NPR**
**Cell 3-3**	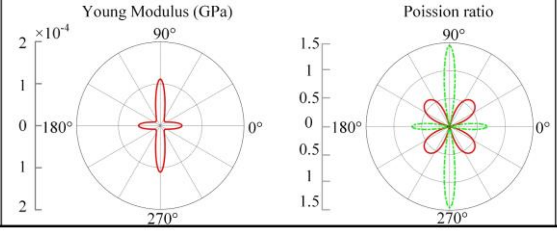	**Anisotropy**	**NPR**

Figure S6. (continued) Elastic properties of basic cells in the Numerical Experiments. Young's modulus curves and Poisson's ratio curves of 17 basic cells (this part includes Cell 2-3 to Cell 3-3).

Cell	Elastic properties	Anisotropy/ Isotropy	PPR/ NPR
**Cell 3-4**	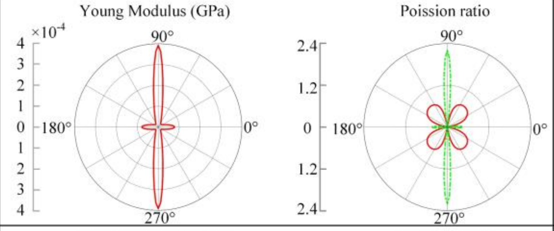	**Anisotropy**	**NPR**
**Cell 3-5**	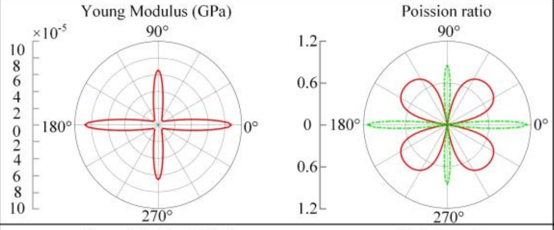	**Anisotropy**	**NPR**
**Cell 3-6**	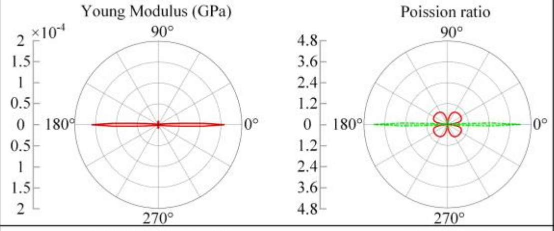	**Anisotropy**	**NPR**
**Cell 4-1**	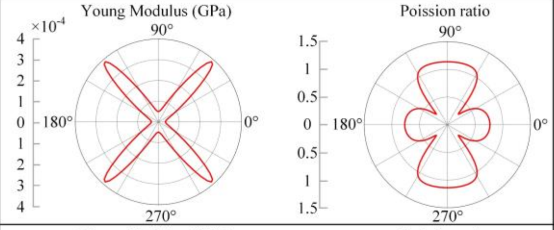	**Anisotropy**	**PPR**
**Cell 4-2**	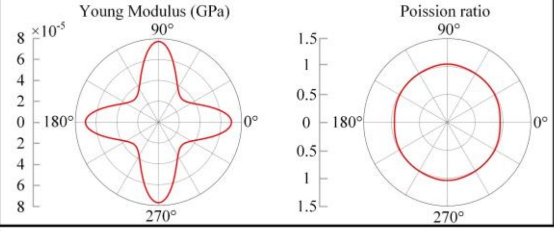	**Anisotropy**	**PPR**

Figure S6. (continued) Elastic properties of basic cells in the Numerical Experiments. Young's modulus curves and Poisson's ratio curves of 17 basic cells (this part includes Cell 3-4 to Cell 4-2).

Cell	Elastic properties	Anisotropy/ Isotropy	PPR/ NPR
**Cell 4-3**	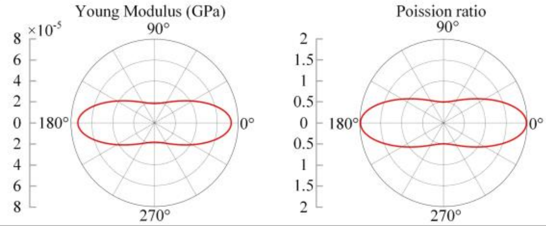	**Anisotropy**	**NPR**
**Cell 4-4**	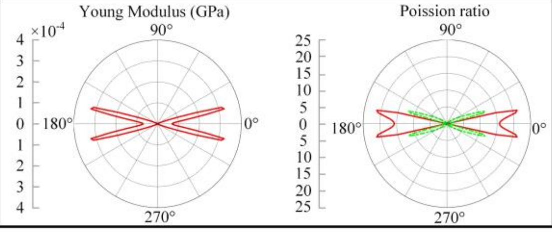	**Anisotropy**	**NPR**

Positive **Negative**

Figure S6. (continued) Elastic properties of basic cells in the Numerical Experiments. Young's modulus curves and Poisson's ratio curves of 17 basic cells (this part includes Cell 4-3 and Cell 4-4).

Group	Cell pairs		
A	Anisotropy & PPR + Anisotropy & NPR：	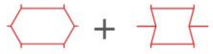	Cell 4-2 + Cell 3-4
B	Anisotropy & PPR + Anisotropy & NPR：	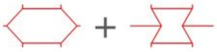	Cell 4-3 + Cell 3-5
C	Anisotropy & PPR + Anisotropy & NPR：	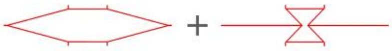	Cell 4-4 + Cell 3-6
D	Anisotropy & NPR + Anisotropy & NPR：	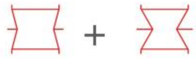	Cell 3-2 + Cell 3-3
E	Isotropy & PPR + Isotropy & NPR：	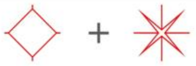	Cell 2-3 + Cell 1-1
F	Isotropy & PPR + Isotropy & NPR：	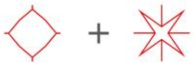	Cell 2-2 + Cell 1-2
G	Isotropy & PPR + Isotropy & NPR：	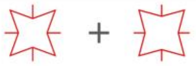	Cell 1-3 + Cell 1-4
H	Isotropy & PPR + Isotropy & PPR：	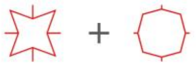	Cell 1-3 + Cell 2-1
I	Isotropy & PPR + Anisotropy & NPR：	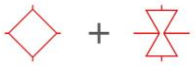	Cell 2-3 + Cell 3-1
J	Anisotropy & PPR + Isotropy & NPR：	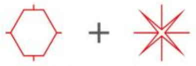	Cell 4-1 + Cell 1-1
K	Isotropy & PPR + Anisotropy & PPR：	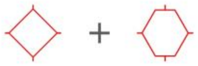	Cell 2-3 + Cell 4-1
L	Anisotropy & NPR + Isotropy & NPR：	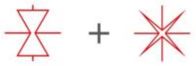	Cell 3-1 + Cell 1-1

Figure S7. 12 cell pairs and pairing details of the basic cells. Cell pairs are generated from the above cells by pairwise combination. Specifically, 17 basic cells with different elastic properties are selected and applied in the generation of 136 cell pairs. By employing the cross combinatorial principle, these cell pairs are further winnowed down to 12 for HRVE construction, including anisotropy-anisotropy (group A to group D), isotropy-isotropy (group E to group H), and isotropy-anisotropy (group I to group L) in terms of Young's modulus; PPR-PPR (group H and group K), NPR-NPR (group D and group L), and PPR-NPR (others) in terms of Poisson's ratio; and the combination of both.

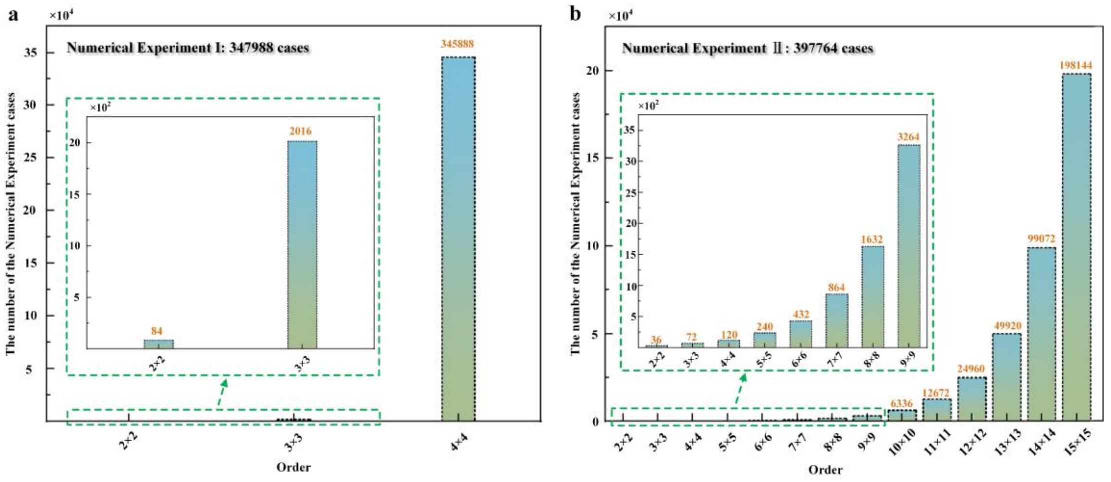

Figure S8. Number distribution of HAMs with different HRVE orders in numerical experiments. The number of HAMs is calculated by all possible assemblies after excluding the repeat geometry (i.e., HRVE geometries are *x*-axisymmetric, *y*-axisymmetric, and rotationally symmetric). In Numerical Experiment I, the HRVE order of the 12 cell pairs is from order 2 × 2 to order 4 × 4, and the total number of HAMs is 347,988 (i.e., 28,999 × 12, where 28,999 is the number of all possible assemblies for each cell pair, $2^4 + 2^9 + 2^{16} = 66{,}064$, subtracted the number of repeated geometries, 37,065). The HRVE order of the cell pair was increased to 15 × 15 order in Numerical Experiment II. Similarly, by excluding the number of repeat geometries, a total of 397,764 HAMs (i.e., 33,147 × 12) are assembled. The number of HAMs at each order in the numerical experiment is given in the figure.

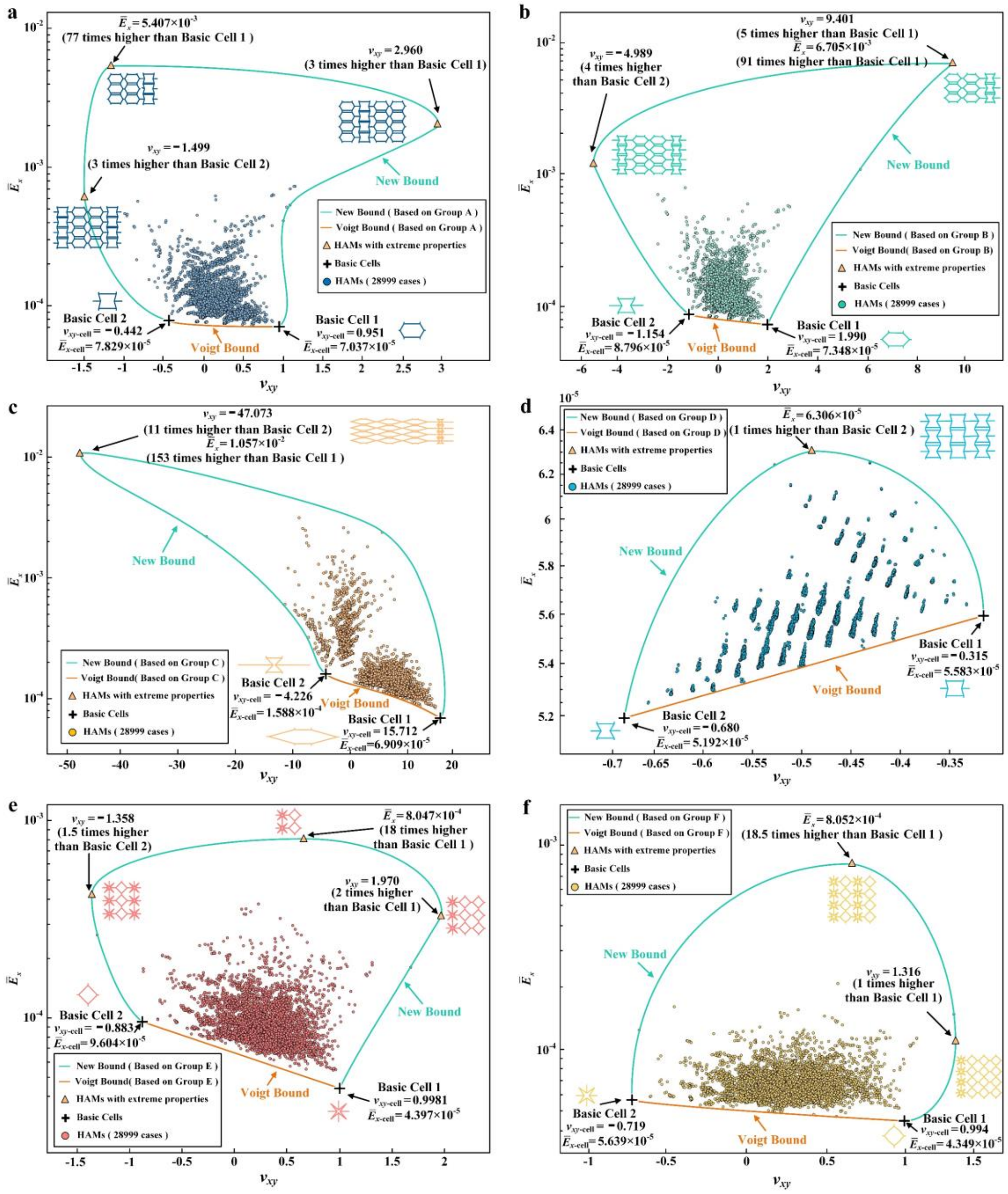

Figure S9. Elastic properties and bounds of 12 group HAMs in Numerical Experiment I. Relative Young's modulus and Poisson's ratio of 12 group HAMs in Numerical Experiment I (this part includes group A to group F, as seen in Figure S9a-f). The figures show the elastic properties of 28,999 HAMs in each group, the elastic properties and geometry of the basic cells, the extreme properties and the corresponding HRVE geometries, and the property bounds. The property bounds for each group include the theoretical bounds (i.e., the Voight bound) used to evaluate the upper limits of the composites and the bounds formed by the HAMs (referred to here as the New bound).

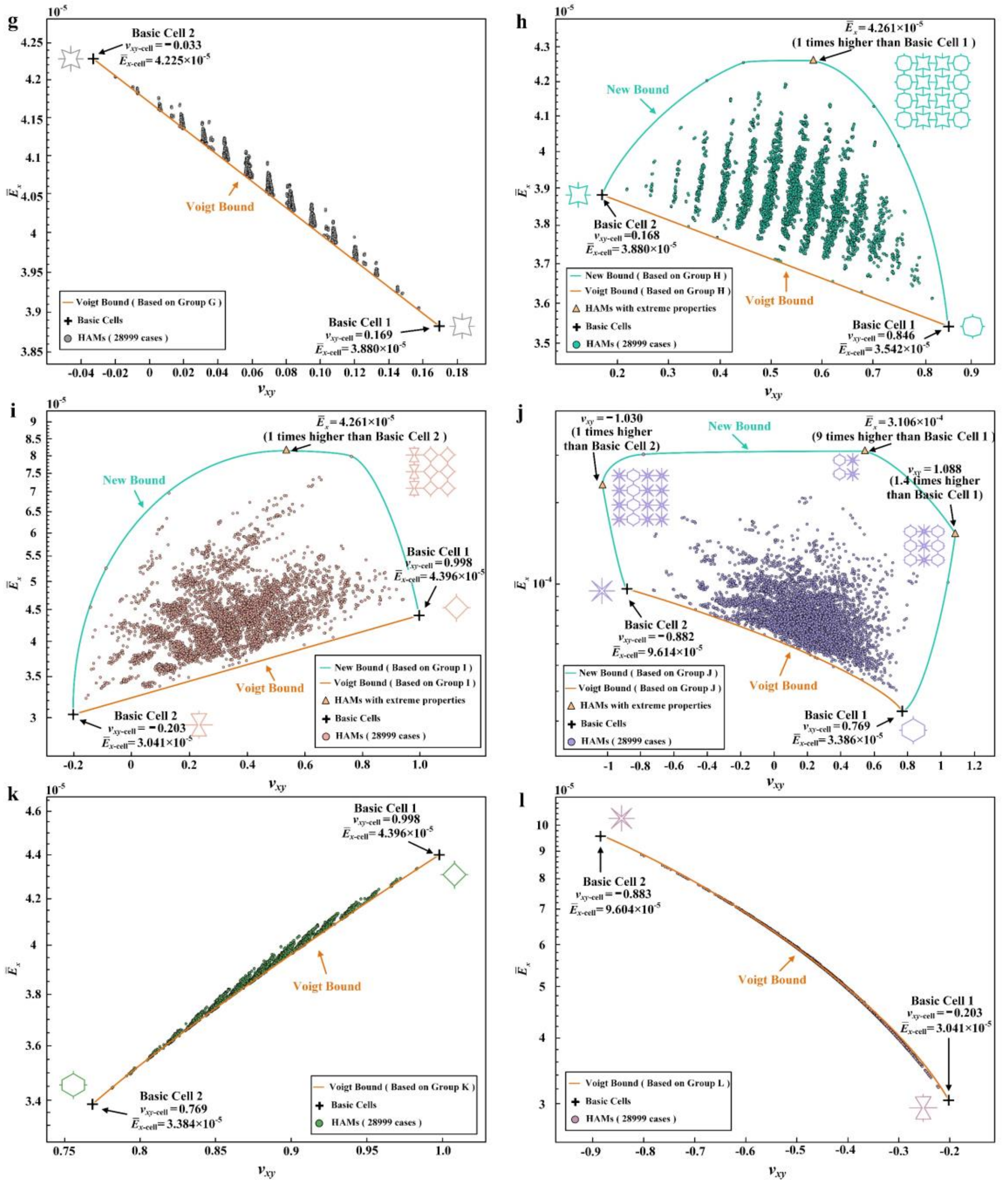

Figure S9. (continued) Elastic properties and bounds of 12 group HAMs in Numerical Experiment I. Relative Young's modulus and Poisson's ratio of 12 group HAMs in Numerical Experiment I (this part includes group G to group L, as seen in Figure S9g-l).

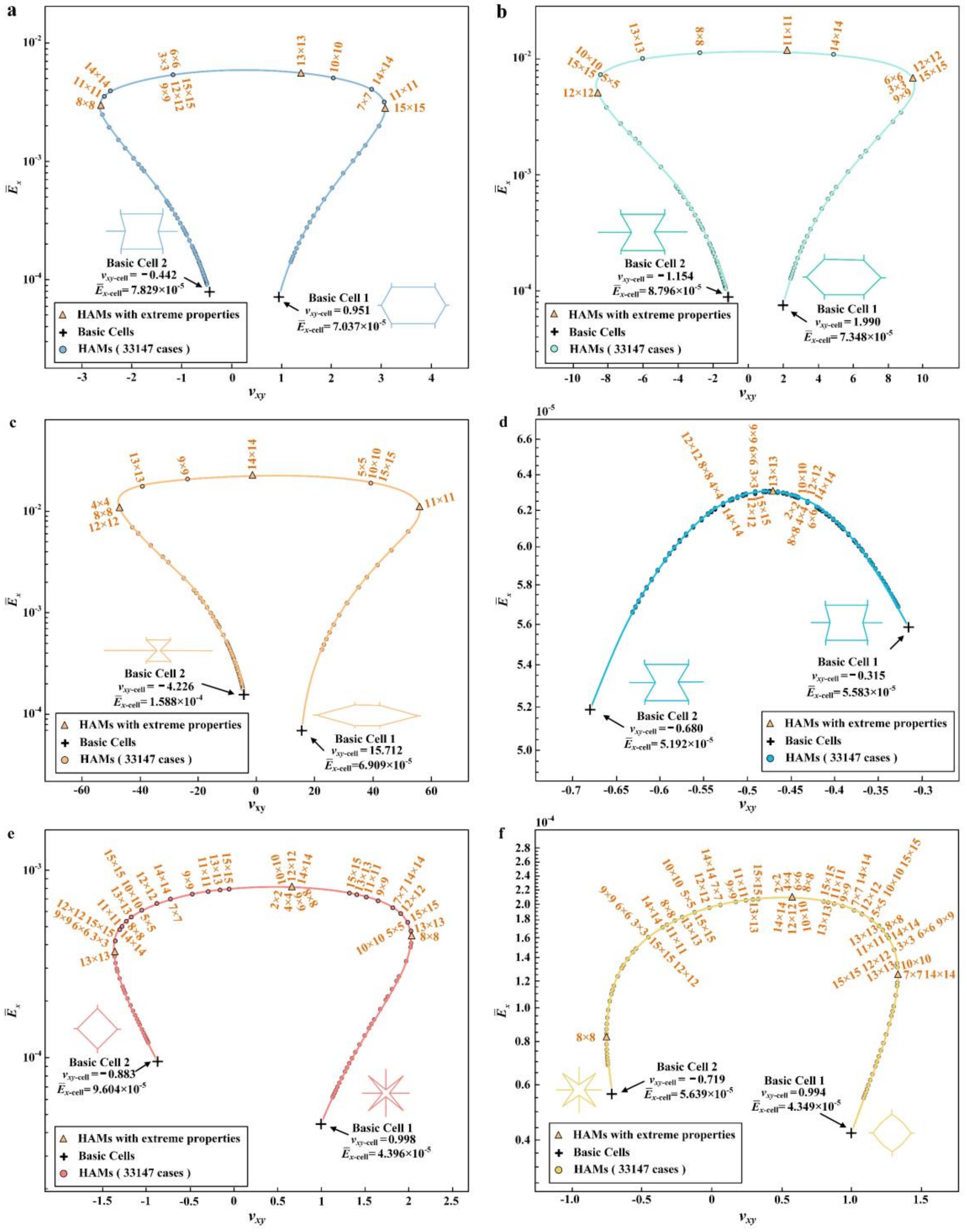

Figure S10. Elastic properties of HAMs in Numerical Experiment II and HRVE order of HAMs with excellent property. Relative Young's modulus and Poisson's ratio of 12 group HAMs in Numerical Experiment II (this part includes group A to group F, as seen in Figure S10 a-f). The figure shows the elastic properties of 33147 HAMs in each group, the geometry and elastic properties of the basic cells, and the HRVE order corresponding to some HAMs with excellent property. There are some obvious features of these HRVE orders among different groups: (1) the HRVE order of HAMs with extreme properties is not the largest order set in Numerical Experiment II; (2) such HRVE orders are not the same.

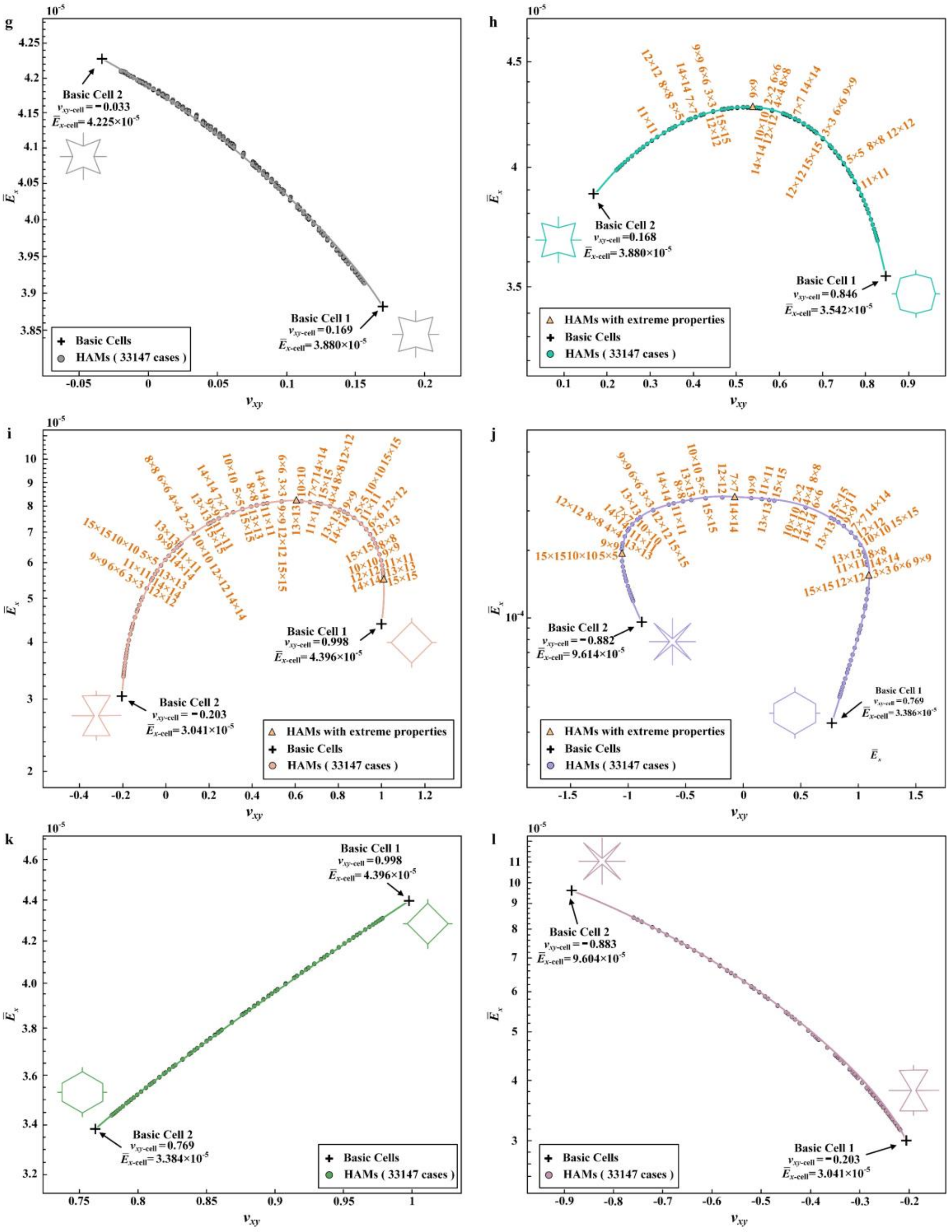

Figure S10. (continued) Elastic properties of HAMs in Numerical Experiment II and HRVE order of HAMs with excellent property. Relative Young's modulus and Poisson's ratio of 12 group HAMs in Numerical Experiment II (this part includes group G to group L, as seen in Figure S10 g-l).

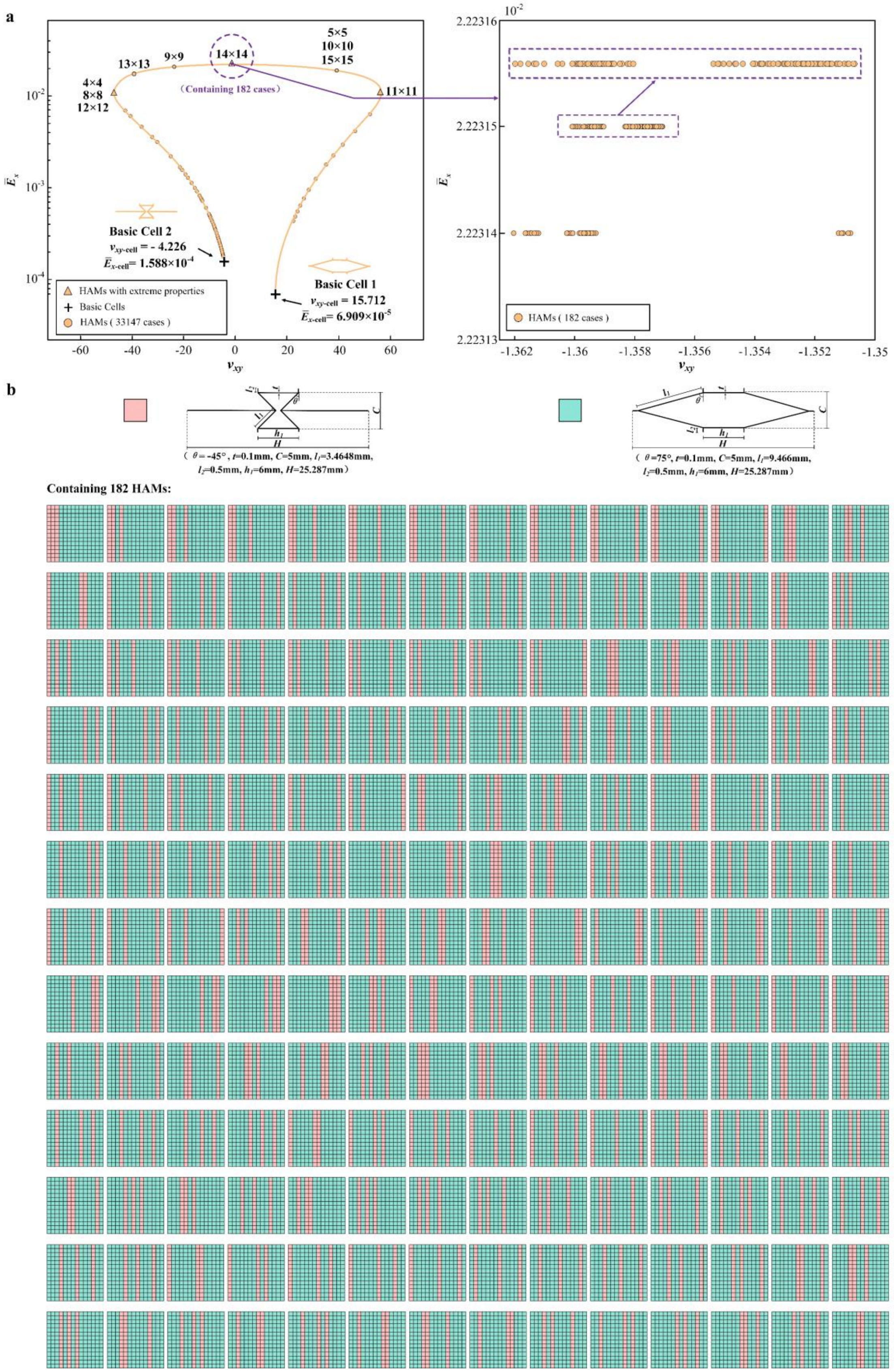

Figure S11. In-depth analysis of the point representing the maximum relative Young's modulus in Figure 2c. a) The point representing the maximum relative Young's modulus observed in Figure 2c (i.e., the maximum relative Young's modulus point in group C) is magnified

in the right panel. **b)** Detailed geometric parameters of the basic cell and the HRVE geometry of the 182 HAMs in the enlarged region. In fact, since the slight property variations ($\bar{E}_x$: $2.22314 \times 10^{-2} \sim 2.22316 \times 10^{-2}$; v_{xy}: $-1.362 \sim -1.35$) resulted from simulation errors, we could consider that the elastic properties of these 182 HAMs with different geometries are identical. Meanwhile, the HRVEs of these 182 HAMs have the same row cell ratio. This phenomenon supports the conclusion that the elastic properties of HAMs are correlated with the row cell ratio rather than with the arrangement sequence of the cells in HRVE.

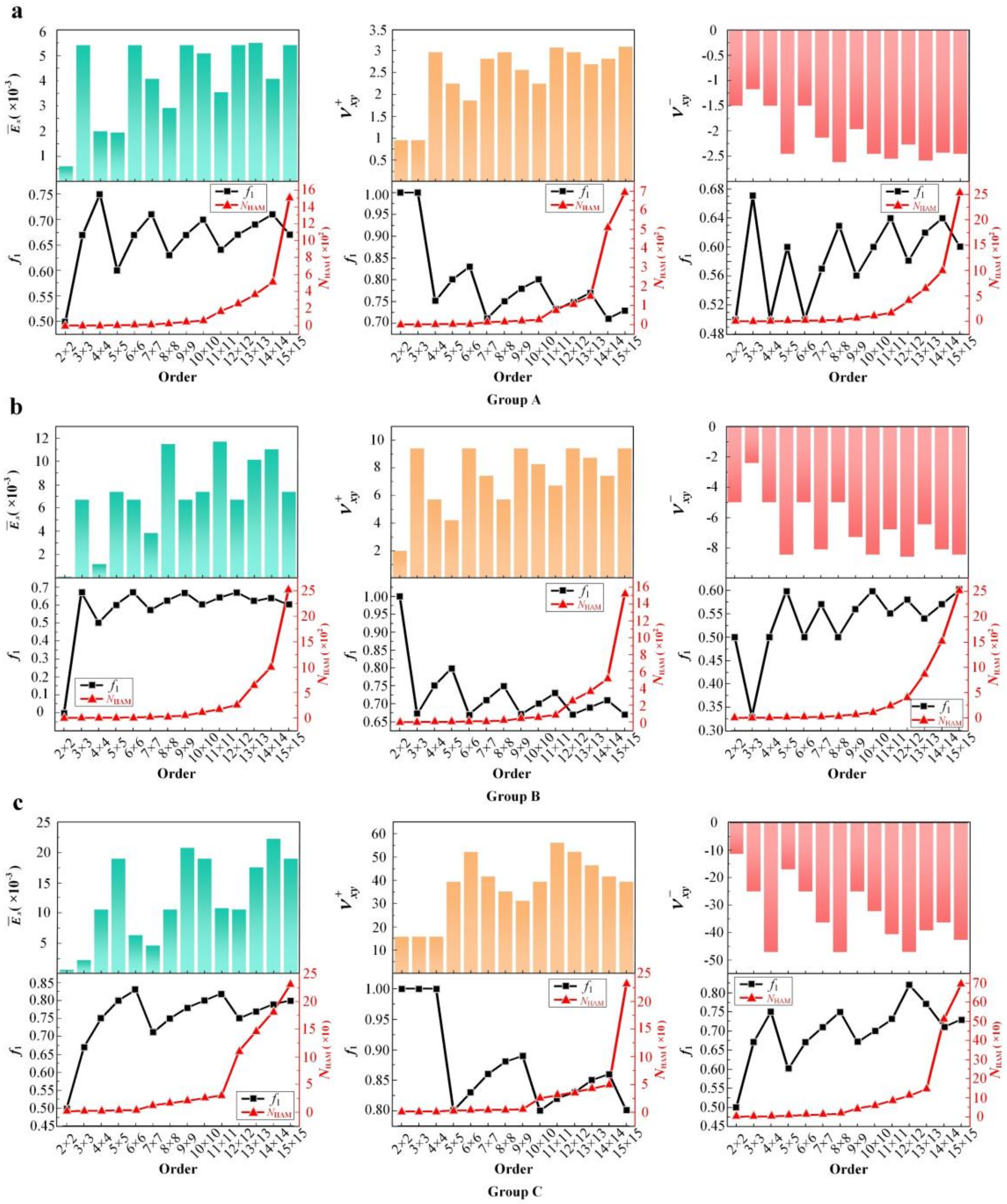

Figure S12. Extremes of elastic properties of HAMs, the length ratio of the Basic Cell 1 and the number of HAMs with extreme properties at each order. Extremes of relative Young's modulus and Poisson's ratio at each order for 9 group HAMs with enhanced elastic properties in Numerical Experiment II. The figure shows the number of HAMs with extreme properties, N_{HAM},

and the length ratio of Basic Cell 1 to the whole HRVE row in the loaded direction, f_1 (This part includes group A to group C, as seen in Figure S12 a-c). Since the HAMs in numerical experiments are assembled from two types of cells, this length ratio of the basic cells could be employed to characterize the HRVE row cell ratio. The one-to-one correspondence between the property extremes, the HRVE row cell ratio, and the number of HAMs verifies the conclusion that the elastic properties of HAMs are correlated with the HRVE row cell ratio.

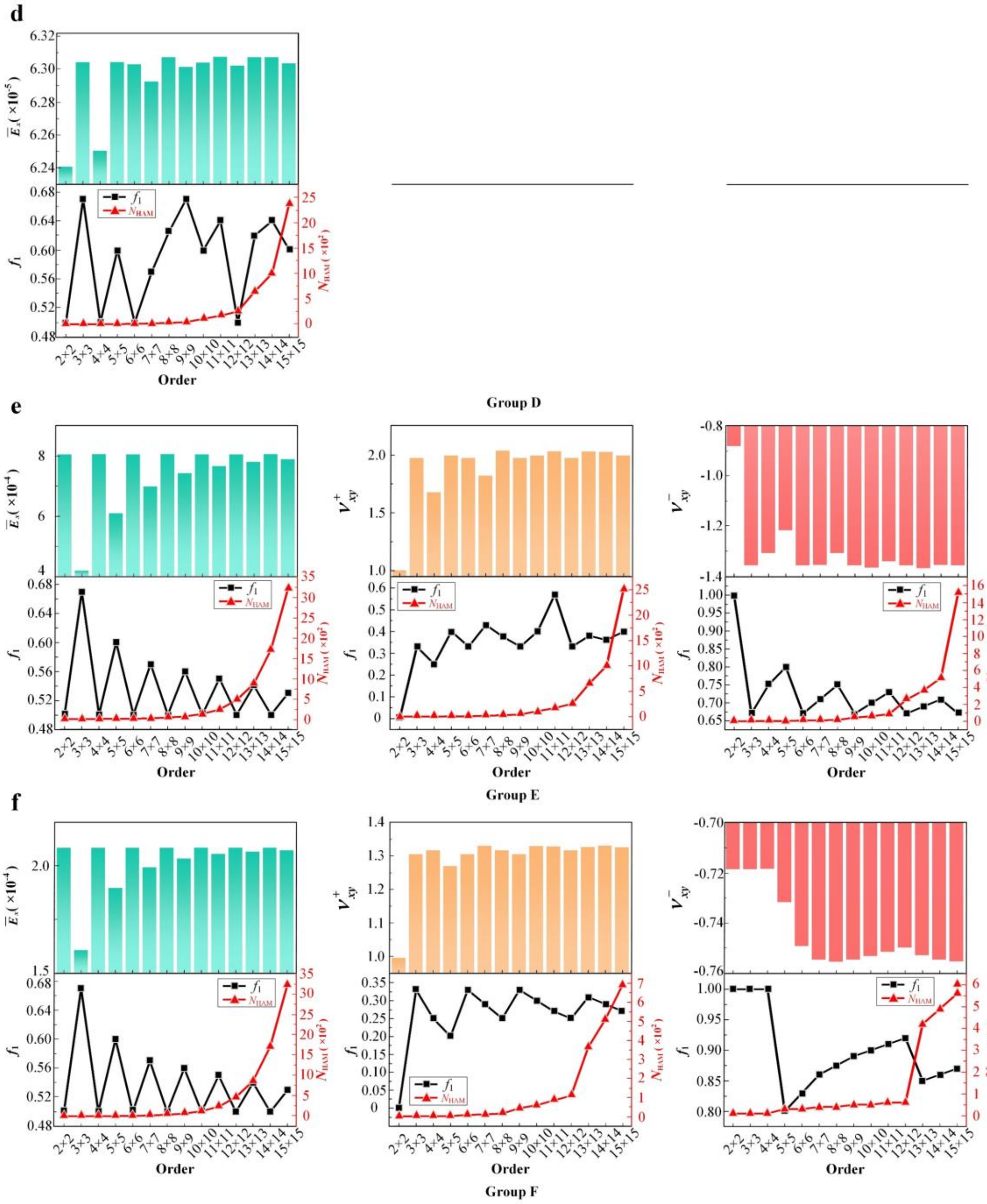

Figure S12. (continued) Extremes of elastic properties of HAMs, the length ratio of the Basic Cell 1 and the number of HAMs with extreme properties at each order. Extremes of relative Young's modulus and Poisson's ratio at each order for 9 group HAMs with enhanced elastic properties in Numerical Experiment II. Meanwhile, the figure shows the number of HAMs with extreme properties and the length ratio of Basic Cell 1 to the whole HRVE row in the loaded

direction (this part includes group D to group F, as seen in Figure S12 d-f).

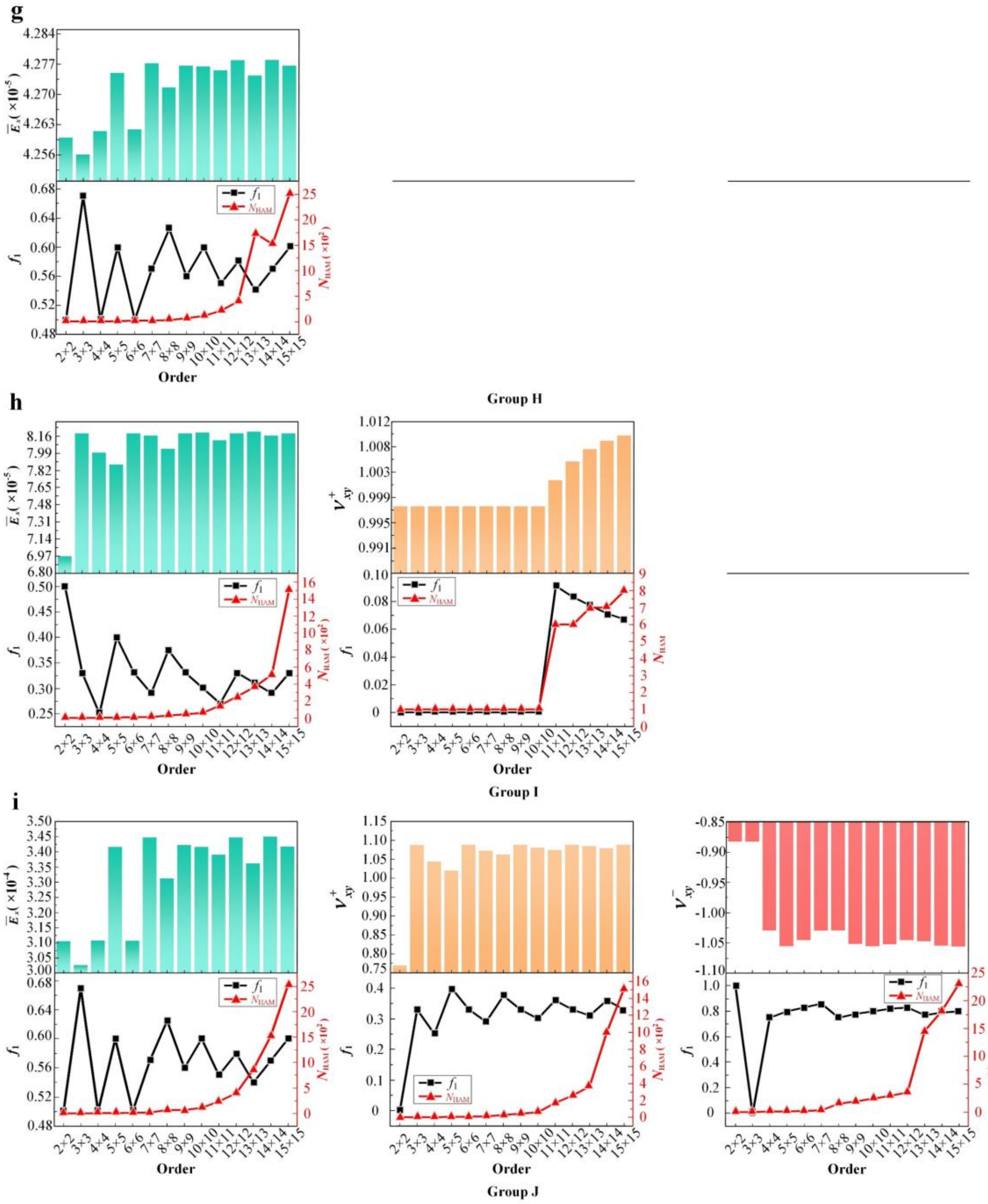

Figure S12. (continued) Extremes of elastic properties of HAMs, the length ratio of the Basic Cell 1 and the number of HAMs with extreme properties at each order. Extremes of relative Young's modulus and Poisson's ratio at each order for 9 group HAMs with enhanced elastic properties in Numerical Experiment II. Meanwhile, the figure shows the number of HAMs with extreme properties and the length ratio of Basic Cell 1 to the whole HRVE row in the loaded direction (this part includes group H, group I, and group J, as seen in Figure S12 g-i).

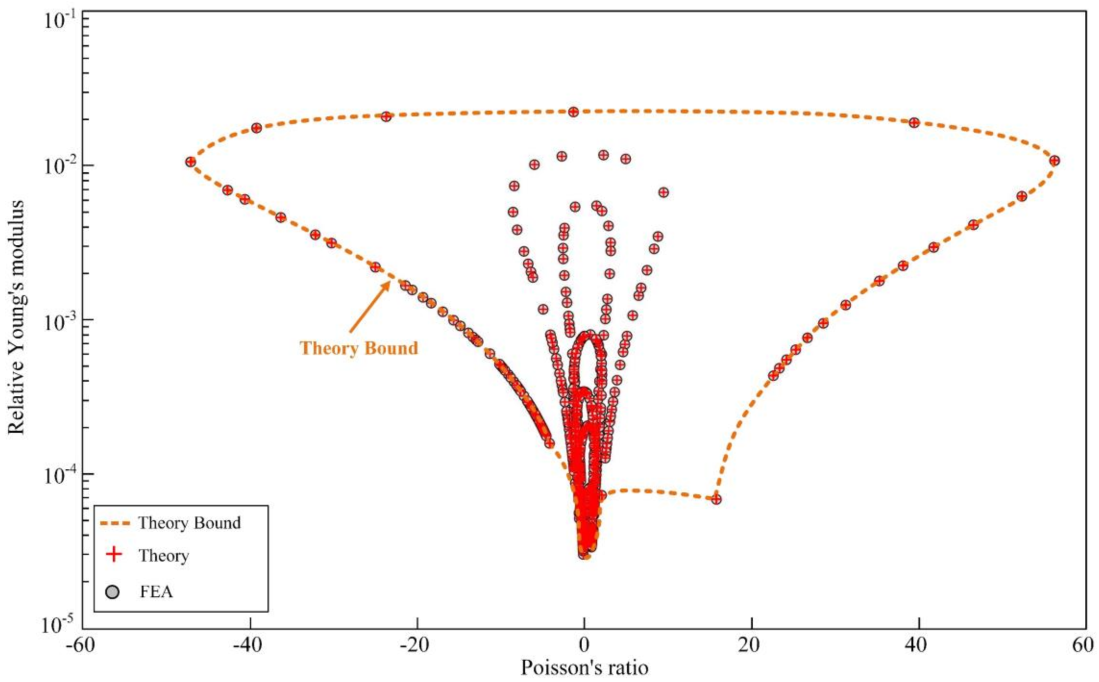

Figure S13. **Theoretical predictions and FEA data of elastic properties of HAMs.** The figure shows the theoretical predictions and FEA results of the elastic properties of 397,764 HAMs in Numerical Experiment II. The good agreement between them indicates that the elastic properties of HAMs can be accurately evaluated by our proposed theoretical model.

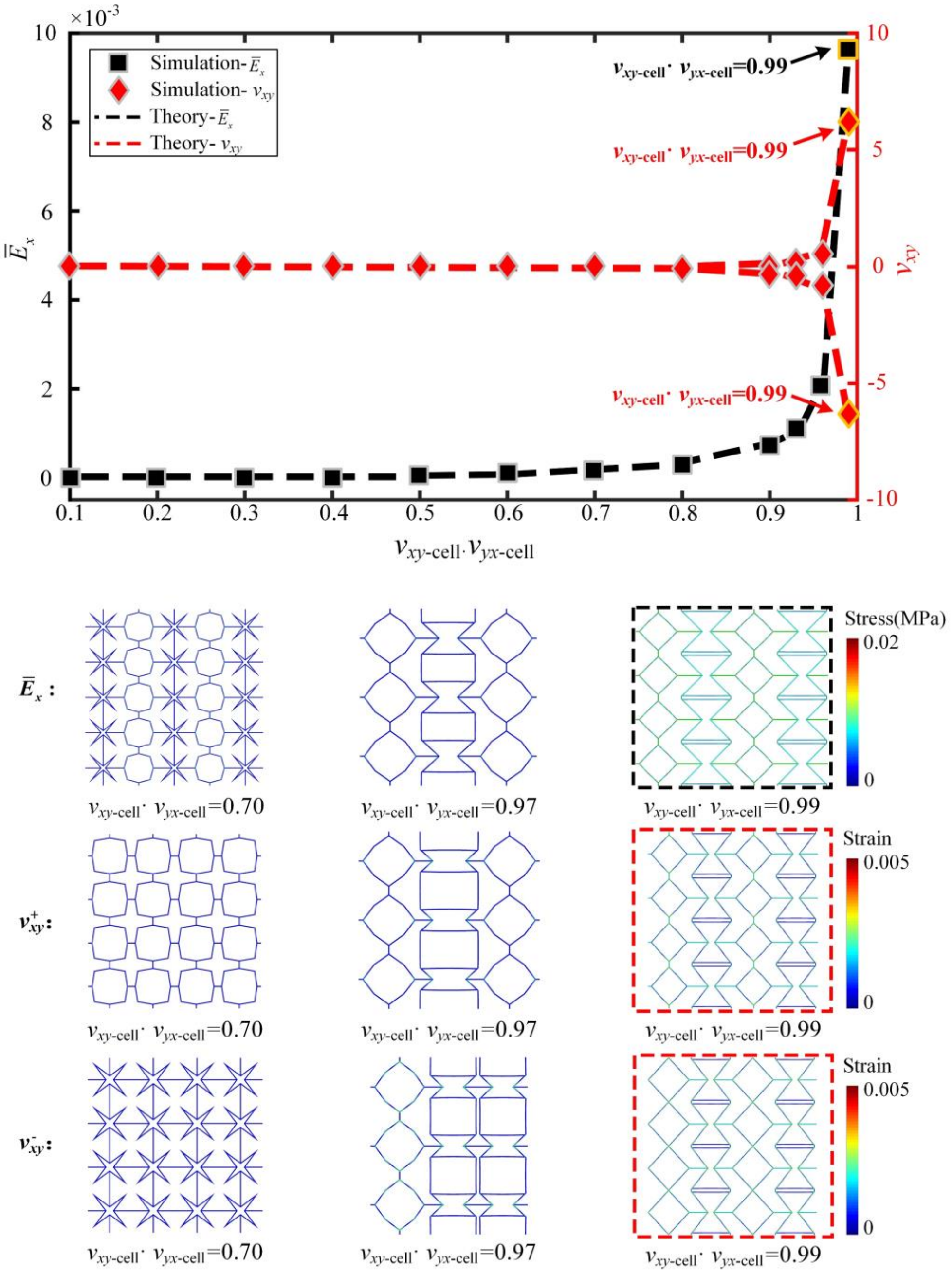

Figure S14. **Elastic properties of HAMs versus the Poisson's ratio product of basic cells.** For all simulation cases presented in this figure, materials are characterized by the HRVE applied with PBC. To determine the relationship between the Poisson's ratio product of cells (i.e., $\nu_{yx-\text{cell}} \cdot \nu_{xy-\text{cell}}$) and the elastic properties of the HAMs, several cells with different Poisson's ratio products were assembled based on the optimal row cell ratio (theoretical calculation). The geometry of the cells was obtained by adjusting the geometric parameters of the classical topology (**Figure S5**). Considering the minimum size of HRVE and the optimal row cell ratio, the number of different cells in HRVEs is determined. The trend of the material properties confirms the validity of the condition 1 (i.e., $1 - \nu_{yx}^{m,n}\nu_{xy}^{m,n} = 0$) in the theoretical model, i.e., the elastic properties of the HAMs would be better as the Poisson's ratio product of the basic cells approaches 1.

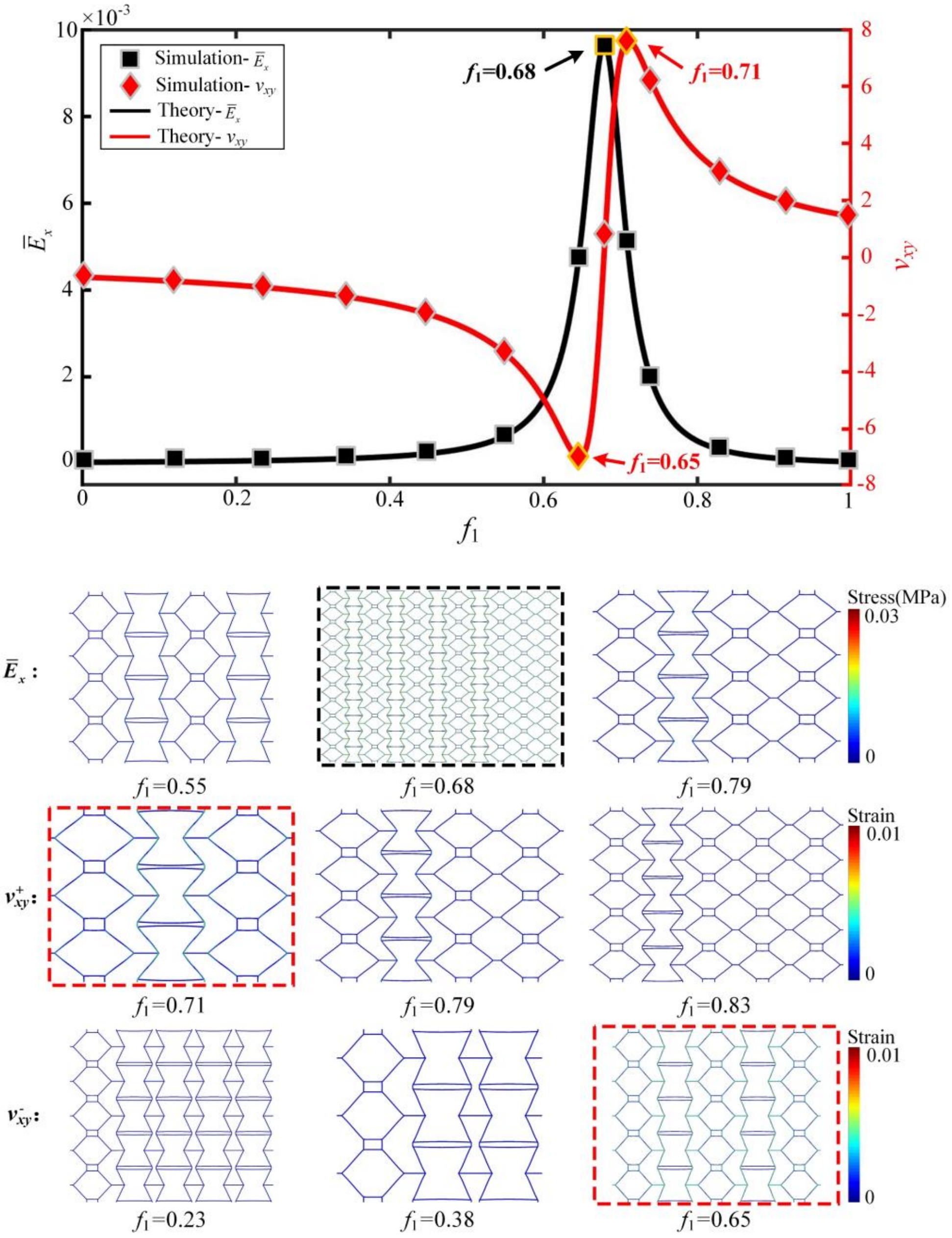

Figure S15. **Elastic properties of HAMs versus the row cell ratio in HRVE.** As described above, the HRVE row cell ratio could be characterized by the length ratio of the basic cells (i.e., the length ratio of the PPR cell, f_1). Therefore, to determine how the HRVE row cell ratio affects the elastic properties of HAMs, HRVEs with different cell length ratios were constructed by employing a pair of cells with positive and negative Poisson's ratios. To make this effect more pronounced, the geometry of the cells with Poisson's ratio products close to 1 (i.e., Poisson's ratio product: 0.996 for the PPR cell, 0.993 for the NPR cell) was obtained by optimizing key geometric parameters. The figure shows the evolution of theoretical predictions and simulation data of the material properties with f_1. The existence of optimal length ratios indicates that the extreme properties have uniquely matched HRVE row cell ratios, and such cell ratio can be achieved by changing the cell number in the HRVE with the aid of the theoretical model (i.e., condition 2, $\sum_{n=1}^{N} \delta_n v_{yx}^{m,n} = 0$).

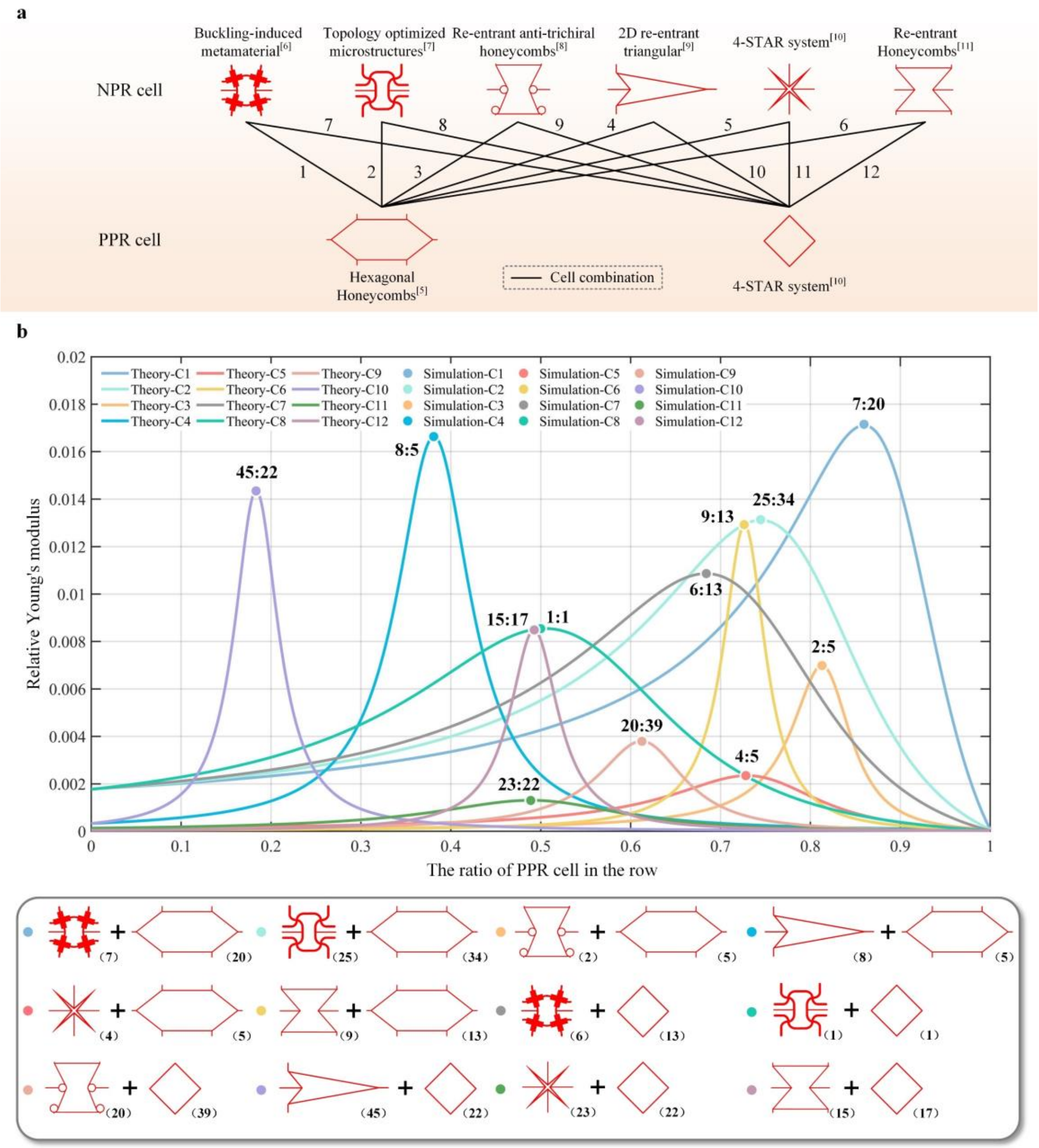

Figure S16. **Geometric generality of the criteria. a)** Some reported NPR and PPR materials, including buckling-induced metamaterial,[6] topology optimized microstructures,[7] re-entrant anti-trichiral honeycombs,[8] 2D re-entrant triangular,[9] 4-STAR system,[10] re-entrant honeycombs,[11] and hexagonal honeycombs,[5] were used to demonstrate the geometric generality of the design criteria. 12 Cell combinations were formed according to one NPR cell paired with one PPR cell (a requirement of the criteria for the basic cell). **b)** Relative Young's modulus of the HAMs versus the length ratio of PPR cells in the HRVE row. The figure shows the theoretical prediction data of the material properties for 12 cell combinations (C1 to C12), the FEA simulation data for HAMs with near-optimal row cell ratios (for Young's modulus) in each combination, and the number of different cells in the HRVE corresponding to these HAMs (i.e., the number of NPR cells and PPR cells). The good agreement between the theoretical and simulated data indicates that our proposed criteria are also applicable to the design of extreme material properties for these cell combinations with different geometries (**Figure S17**), thereby explaining the geometric generality of the criteria.

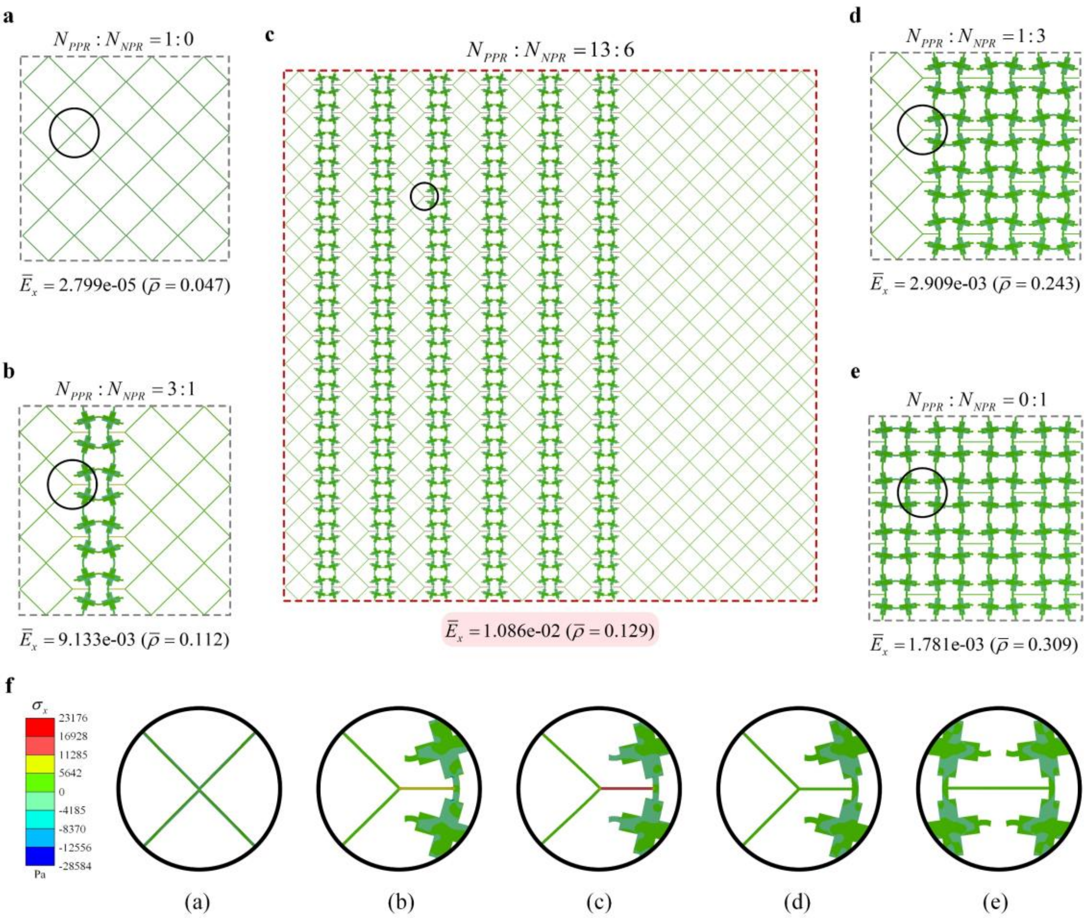

Figure S17. **Mechanical response of HAMs with different row cell ratios.** Stress distribution in the loaded direction (σ_x), relative Young's modulus ($\bar{E}_x$), and relative density ($\bar{\rho}$) for HAMs with different row cell ratios (form **a)** to **e)**) assembled by C7 (**Figure S16**). The color scale in **f)** provides a measure of stress. As shown in **c)**, HAM with a suitable row cell ratio exhibits more superior Young's modulus at a lower relative density. Importantly, the application of the generic design criteria allows us to directly obtain such row cell ratios for cell combinations with different geometries.

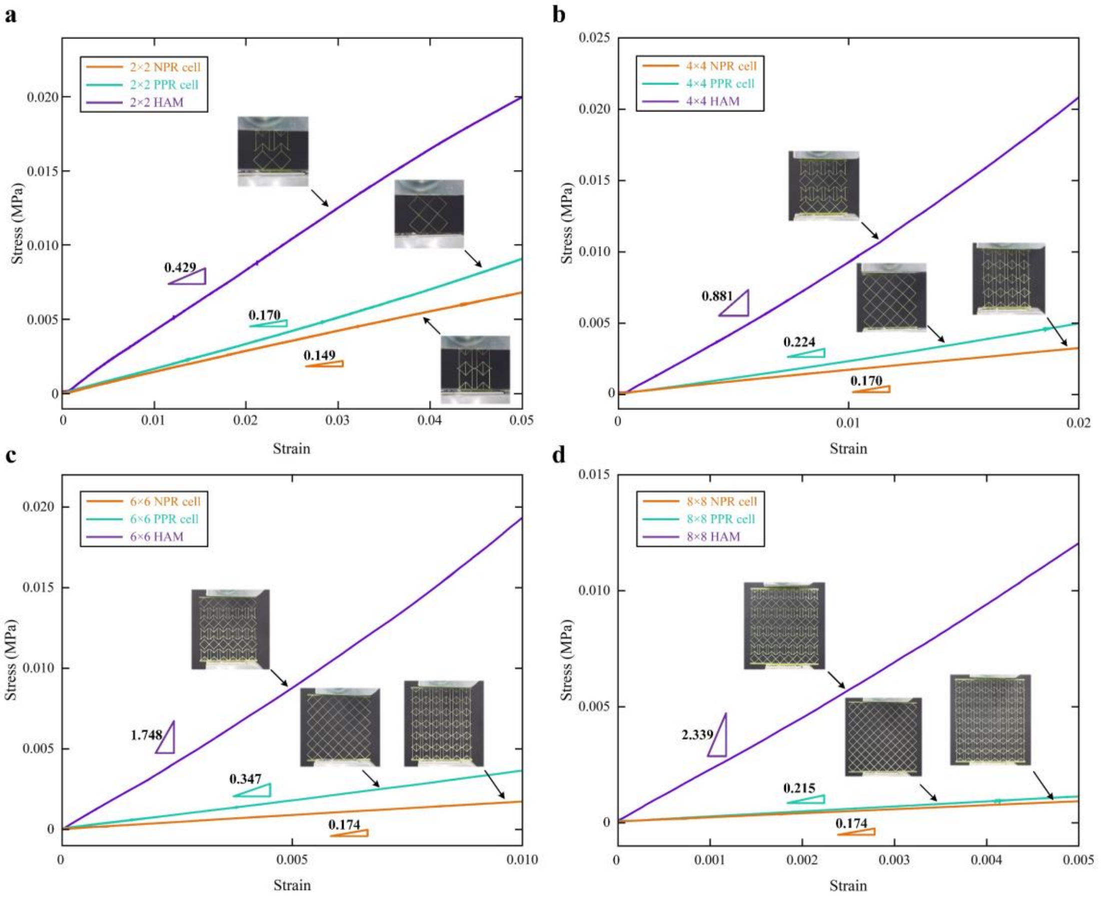

Figure S18. **Experimental stress-strain curves for material samples with different periodic arrangement orders (from 2×2 to 8×8). a)** 2×2 material samples. **b)** 4×4 material samples. **c)** 6×6 material samples. **d)** 8×8 material samples (including HAMs and materials consisting of periodic arrangement of a single NPR cell or PPR cell). The figures provide the Young's modulus for each sample.

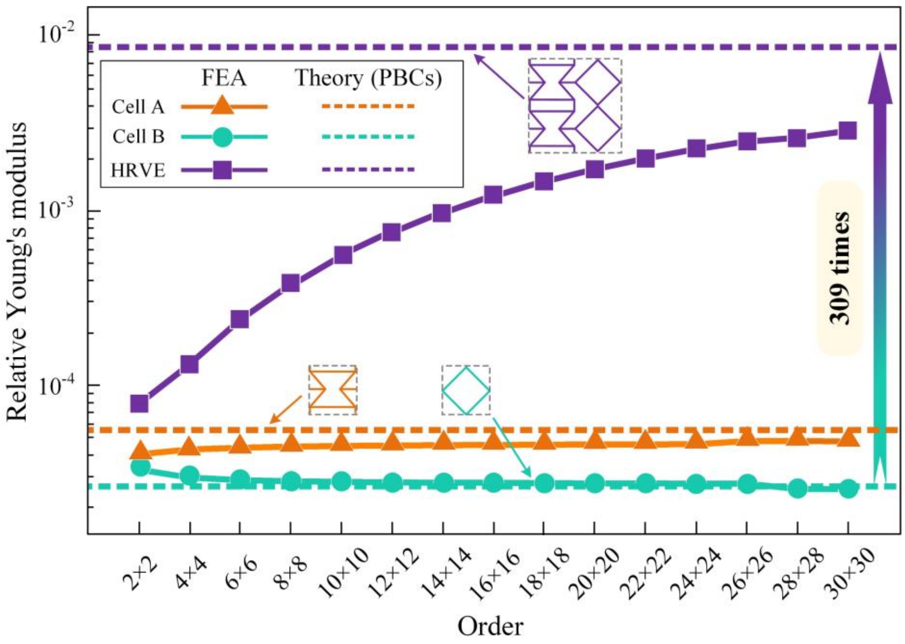

Figure S19. **Plot of relative Young's modulus versus order for HAMs and basic cells.** The Young's modulus of HAMs shows a tendency to converge to the ideal properties of the material under PBCs, while the properties of the material formed by a single cell have converged to their respective such values. In the effect of PBCs, HAM was enhanced by 309 times in Young's modulus compared to PPR cell (i.e., Cell B).

Table S1. Geometric parameters (based on the topology 2 and 3 in numerical experiments, as shown in Figure S5), relative Young's modulus, and Poisson's ratio product of basic cells used in mechanical testing.

Basic cells	Geometric parameters						Relative Young's modulus ($\bar{E}_x$)	Poisson's ratio product ($\nu_{xy}\nu_{yx}$)
PPR cell	b(mm)	l_1(mm)	C(mm)	t(mm)	α(°)		2.799e-5	0.998
	3.9	0.000	7.80	0.13	180.0			
NPR cell	L(mm)	l_1(mm)	C(mm)	t(mm)	θ(°)	l(mm)	5.580e-5	0.992
	9.1	0.975	5.85	0.13	44.4	8.84		

Table S2. Relative density, geometric parameters (based on the topology 3 and 4 in numerical experiments, as shown in Figure S5), cell number in HRVE, relative Young's modulus, and $\bar{E}_x/\bar{\rho}$ of HAMs with extreme Young's modulus.

Relative density of HAMs ($\bar{\rho}$)	Basic cells	Geometric parameters of cells						Cell number	Relative Young's modulus ($\bar{E}_x$)	$\bar{E}_x/\bar{\rho}$
		L(mm)	l_1(mm)	C(mm)	t(mm)	θ(°)	l(mm)			
0.00851	PPR cell	183.2870	0.3405	18.3190	0.08	83.5420	21.1570	10	0.00792	0.9307
	NPR cell	34.5000	6.8750	5.2500	0.08	40.7790	9.9728	1		
0.01009	PPR cell	184.6510	0.3770	15.2460	0.08	84.8433	15.5960	10	0.00967	0.9584
	NPR cell	35.2700	7.6410	0.7180	0.08	41.6484	21.6128	1		
0.01407	PPR cell	129.4590	3.4895	8.0210	0.10	85.0000	38.8870	10	0.01275	0.9062
	NPR cell	40.0000	6.7910	1.4180	0.10	45.7805	14.6507	1		
0.01559	PPR cell	146.2310	0.8325	11.3350	0.10	84.9355	18.4520	20	0.01461	0.9371
	NPR cell	30.5260	0.3000	12.4000	0.10	29.2677	10.4977	1		
0.01682	PPR cell	142.9410	0.5810	10.8380	0.10	85.0000	20.0000	13	0.01595	0.9483
	NPR cell	38.2030	4.2030	3.5940	0.10	40.0444	11.6942	1		
0.02008	PPR cell	109.3460	0.3125	9.3750	0.10	84.4633	12.6208	20	0.01902	0.9472
	NPR cell	27.7520	4.1980	1.6040	0.10	23.8119	12.6650	1		
0.02243	PPR cell	113.6290	0.7515	7.4970	0.10	85.2504	21.6720	17	0.02121	0.9456
	NPR cell	32.2070	2.3395	4.3210	0.10	34.9848	13.3638	1		
0.02509	PPR cell	86.3340	0.5360	6.9280	0.10	84.6219	12.8060	16	0.02345	0.9346
	NPR cell	30.5760	2.6880	2.6240	0.10	32.3196	2.9530	1		
0.02836	PPR cell	106.7210	0.8480	8.3040	0.14	84.8621	14.9190	21	0.02662	0.9386
	NPR cell	22.7500	3.2730	3.4540	0.14	26.3090	9.2439	1		
0.03025	PPR cell	116.9960	0.4350	9.1300	0.15	84.6162	20.7130	10	0.02863	0.9464
	NPR cell	22.6390	3.9835	2.0330	0.15	42.8397	12.3855	1		
0.03874	PPR cell	70.3930	1.4635	5.0730	0.15	84.1480	21.3580	12	0.03488	0.9003
	NPR cell	28.4770	2.7975	2.4050	0.15	36.3280	12.7445	1		
0.04702	PPR cell	65.6660	1.2675	4.4650	0.16	84.8145	17.2320	16	0.04300	0.9145
	NPR cell	23.8040	2.5590	1.8820	0.16	30.5644	13.3731	1		

Table S3. $\bar{E}_x/\bar{\rho}$ of HAM and other reported mechanical metamaterials in relative density $\bar{\rho} = 0.03$. [12-19]

Materials	Microlattices[12]	Nanolattice[13]	Shellular[14]	Cubic foam[15]	3D Plate-Lattices[16]	Lattice structures[17]	CMC based metamaterials[18]	Tube-in-tube structure[19]	HAMs (this work)
$\bar{E}_x/\bar{\rho}$ ($\bar{\rho} = 0.03$)	0.170	0.060	0.083	0.710	0.500	0.500	0.440	0.021	0.946

Legends for 4 Supplementary Movies

Movie S1. Testing for the property enhancement effects of assembly design. Load-bearing capacity testing for 8×8 HAM, 8×8 Cell A and 8×8 Cell B (250-g weight for the HAM, and 50-g weight for the cells).

Movie S2. Uniaxial tensile tests of 10×10 HAM.

Movie S3. Uniaxial tensile tests of 10×10 PPR cell.

Movie S4. Uniaxial tensile tests of 10×10 NPR cell.

References

[1] L. J. Gibson, M. F. Ashby, *Cellular solids : structure and properties*, Cambridge university press, **1997**.

[2] Z. H. Xia, C. W. Zhou, Q. L. Yong, X. W. Wang, *Int. J. Solids. Struct.* **2006**, 43, 266.

[3] O. Sigmund, *Int. J. Solids. Struct.* **1994**, 31, 2313.

[4] J. N. Grima, R. Gatt, A. Alderson, K. E. Evans, *Mol Simul*. **2005**, 31, 925

[5] L. J. Gibson, M. F. Ashby, G. S. Schajer, C. I. Robertson, *P. Roy. Soc. Lond. a. Mat.* **1982**, 382, 25.

[6] A. Ghaedizadeh, J. H. Shen, X. Ren, Y. M. Xie, *Materials*. **2016**, 9.

[7] A. Clausen, F. W. Wang, J. S. Jensen, O. Sigmund, J. A. Lewis, *Adv. Mater.* **2015**, 27, 5523.

[8] A. Alderson, K. L. Alderson, G. Chirima, N. Ravirala, K. M. Zied, *Compos. Sci. Technol.* **2010**, 70, 1034.

[9] U. D. Larsen, O. Sigmund, S. Bouwstra, *J Microelectromech S*. **1997**, 6, 99.

[10] J. N. Grima, R. Gatt, A. Alderson, K. E. Evans, *Mol. Simulat.* **2005**, 31, 925.

[11] I. G. Masters, K. E. Evans, *Compos. Struct.* **1996**, 35, 403.

[12] X. Y. Zheng, H. Lee, T. H. Weisgraber, M. Shusteff, J. DeOtte, E. B. Duoss, J. D. Kuntz, M. M. Biener, Q. Ge, J. A. Jackson, S. O. Kucheyev, N. X. Fang, C. M. Spadaccini, *Science* **2014**, 344, 1373.

[13] L. R. Meza, S. Das, J. R. Greer, *Science* **2014**, 345, 1322.

[14] S. C. Han, J. W. Lee, K. Kang, *Adv. Mater.* **2015**, 27, 5506.

[15] J. B. Berger, H. N. G. Wadley, R. M. Mcmeeking, *Nature* **2017**, 543, 533.

[16] T. Tancogne-Dejean, M. Diamantopoulou, M. B. Gorji, C. Bonatti, D. Mohr, *Adv. Mater.* **2018**, 30.

[17] Y. Q. Wang, O. Sigmund, *Extreme Mech. Lett.* **2020**, 34.

[18] B. W. Deng, G. J. Cheng, *Mater. Horiz.* **2021**, 8, 987.

[19] J. C. Ye, L. Liu, J. Oakdale, J. Lefebvre, S. Bhowmick, T. Voisin, J. D. Roehling, W. L. Smith, M. R. Ceron, J. Van Ham, L. B. B. Aji, M. M. Biener, Y. M. Wang, P. R. Onck, J. Biener, *Nat. Mater.* **2021**, 20, 1498.
